# Sex-Dependent Lipidomic Remodeling in Plasma and Feces Following High-Fat Diet Intake in Adult Rats

**DOI:** 10.3390/nu18183014

**Published:** 2026-09-15

**Authors:** Sofía González-las Heras, Ruth Forsten, Karla Rio-Aige, Margarida Castell, Adele Costabile, Silvia Melgar, Francisco J. Pérez-Cano, Malén Massot-Cladera, Maria José Rodríguez-Lagunas

**Affiliations:** 1 Physiology Section, Department of Biochemistry and Physiology, Faculty of Pharmacy and Food Science, University of Barcelona (UB), 08028 Barcelona, Spain; sofi.gonzalez29@ub.edu (S.G.-l.H.); ruth.forsten@ub.edu (R.F.); rioaigekarla@ub.edu (K.R.-A.); margaridacastell@ub.edu (M.C.); franciscoperez@ub.edu (F.J.P.-C.); mjrodriguez@ub.edu (M.J.R.-L.); 2 Nutrition and Food Safety Research Institute (INSA-UB), 08921 Santa Coloma de Gramenet, Spain; 3 Center for Biomedical Research Network for the Physiopathology of Obesity and Nutrition (CI-BEROBN), Instituto de Salud Carlos III, 28029 Madrid, Spain; 4 School of Life and Health Sciences, University of Roehampton (Roe), London SW15 4JD, UK; adele.costabile@roehampton.ac.uk; 5 APC Microbiome Ireland, University College Cork, Cork T12 YT20, Ireland

**Keywords:** high-fat diet, obesity, lipidomics, sex dimorphism, acylcarnitines

## Abstract

Background/Objectives: This study aimed to characterize the effects of high-fat-diet (HFD)-induced metabolic stress on both plasma and fecal lipidomes in rats, and to determine the influence of sex on these responses. Methods: Adult female and male Wistar rats were fed either a standard diet (REF) or HFD (45% lard fat diet) for 12 weeks and metadata were collected over time. Plasma and feces samples were collected and subjected to semi-targeted lipidomic profiling by mass spectrometry. Results: HFD induced significant morphometric and metabolic alterations in both sexes, with a more pronounced metabolic phenotype in males. Plasma lipidomics revealed extensive diet-driven induced remodeling across multiple lipid classes, driven primarily by changes in individual lipid species level rather than total class abundance. Although sex significantly influenced the plasma lipidome in both dietary conditions, the direction of HFD-induced changes in individual lipid species was largely concordant between females and males. In contrast, the fecal lipidome showed limited global remodeling but displayed distinct changes in specific lipid subclasses. Notably, fecal long-chain acylcarnitines (ACar) were consistently increased in HFD animals of both sexes. Fecal ACar 16:0 and ACar 18:1 were positively associated with body weight gain in the HFD group, whereas no significant associations were detected in REF animals. Conclusions: These findings reveal distinct systemic and intestinal lipidomic responses to HFD and highlight the value of integrating fecal lipidomics alongside plasma profiling while considering sex as a fundamental biological variable in metabolic research.

## 1. Introduction

High-fat diets (HFDs) are widely used in preclinical studies to investigate the metabolic disturbances associated with excessive dietary fat intake. Chronic exposure to HFDs promotes profound changes in systemic metabolism, including changes in energy homeostasis, metabolic signaling pathways, and lipid metabolism [1,2]. These alterations are closely reflected in the circulating lipid profile, which integrates the combined effects of dietary intake, hepatic lipid metabolism, and intestinal absorption and utilization [3,4,5].

In addition to systemic lipid modifications, dietary fat intake also affects lipid metabolism within the gastrointestinal tract, where interactions between host physiology and the gut microbiota play a key role. Beyond digestion and absorption, gut microorganisms can modify dietary lipids and generate bioactive lipid species that may enter the host circulation and influence metabolic homeostasis. As a result, the fecal lipidome reflects not only unabsorbed dietary lipids but also host-derived and microbiota-derived metabolites [6,7,8]. Despite its potential to provide insight into diet–host–microbiome interactions, the fecal lipidome remains relatively understudied, and few studies have explored how HFDs remodel fecal lipid profiles alongside systemic lipid alterations. Therefore, a comprehensive characterization of lipid alterations across both systemic and intestinal compartments is essential to better understand the metabolic consequences of high-fat feeding.

Another important aspect that has received increasing attention in metabolic research is the role of biological sex in shaping metabolic responses to dietary challenges. Females and males exhibit significant differences in lipid metabolism, fat distribution, and susceptibility to metabolic disturbances. These differences are influenced by hormonal regulation, energy metabolism, and lipid handling pathways [9,10]. However, many studies have historically been focused on male animals, and sex-specific differences in lipidomic remodeling induced by HFD remain incompletely characterized [11,12].

To comprehensively characterize HFD-induced metabolic alterations, advances in mass-spectrometry-based lipidomics have enabled comprehensive profiling of hundreds of lipid species across multiple lipid classes. This approach provides a detailed view of lipid metabolism, allowing the identification of specific molecular species that may reflect metabolic dysregulation [13]. Several lipid groups, including triacylglycerols, diacylglycerols, ceramides, and phospholipids, have been associated with metabolic disturbances linked to HFD feeding and metabolic disease [14,15,16]. Lipidomic analyses have, therefore, become a powerful tool to investigate how dietary interventions can reshape lipid metabolism across different biological compartments [17].

The aim of the present study was to characterize the lipidomic remodeling induced by HFD consumption in both female and male Wistar rats using a semiquantitative lipidomic approach based on liquid chromatography coupled to mass spectrometry (LC-MS). By simultaneously analyzing plasma and fecal samples, this study sought to investigate diet-induced alterations in systemic and intestinal lipid profiles and to explore potential sex-specific patterns in the lipidomic response to high-fat feeding.

## 2. Materials and Methods

### 2.1. Diets

Two different diets were used: a reference diet (REF) and a high-fat diet (HFD). The REF diet was prepared based on the American Institute of Nutrition (AIN) 93M formulation (TF.94048, Envigo, Indianapolis, IN, USA), indicated for the maintenance of adult, non-reproducing rodents. The HFD diet (TD.06415, Inotiv Tecklad™ Diets, West Lafayette, IN, USA) provided approximately 45% of the total energy from fat, with lard as the primary source (Table S1). In addition to the higher fat content, the two diets differed in the proportions of other dietary components. Specifically, the HFD contained a higher sucrose content (200 vs. 100 g/kg of diet) and slightly higher cellulose content (58 vs. 50 g/kg), as well as differences in the mineral and vitamin mixtures. The HFD also contained calcium phosphate dibasic, which was not included in the REF formulation. Moreover, the fatty acid (FA) composition was approximately 35% saturated, 40% monounsaturated, and 25% polyunsaturated acids.

### 2.2. Animals and Experimental Design

Seven-week-old female and male Wistar rats (14 of each) were purchased from Janvier (Le-Genest-Saint-Isle, France) and housed at the Experimental Animal Facility of the Diagonal Campus located at the Faculty of Pharmacy and Food Science, University of Barcelona. Animals were housed in polycarbonate cages with large fibrous-particle bedding and tissue papers as enrichment under standard conditions of temperature (21 ± 2 °C) and relative humidity (50–55%) with a 12 h light/12 h dark cycle. Water and chow were provided ad libitum. After a one-week acclimatization period, animals were randomly assigned to a dietary group: reference (REF) group fed the REF diet, or HFD group fed the HFD (7 females and 7 males each). The dietary intervention was maintained for 12 weeks.

Body weight (BW) was monitored weekly throughout the study. At the end of week 11, metabolic assessments were performed under fasting conditions, including the determinations of fasting blood glucose (FBG) concentration, an oral glucose tolerance test (OGTT), and fasting insulin (FI) concentration.

After 12 weeks, animals were anesthetized via an intraperitoneal injection of ketamine, 90 mg/kg (Merial Laboratories S.A., Barcelona, Spain), and xylazine, 10 mg/kg (Bayer A.G., Leverkusen, Germany), and euthanized by exsanguination through cardiac puncture. Blood samples were collected in heparinized tubes and centrifuged (1000× *g*, 10 min, 4 °C), followed by plasma collection, and stored at −80 °C until adipokine determination and lipidomic analysis. One fecal pellet was also collected from the colon of each animal and stored at −80 °C for lipidomic analysis. Additionally, morphometric measurements such as body length (nose-to-anus length) and waist circumference (WC) were recorded. BW gain and Lee index, as BW^1/3^/body length × 1000 (g^1/3^/cm), were calculated.

All experimental procedures were reviewed and approved by the Ethics Committee for Animal Experimentation at the University of Barcelona (CEEA/UB 275/23). The minimum number of animals required was estimated based on variability observed in our previous studies on metabolomic measures [18] using the Granmo program (https://www.datarus.eu/aplicaciones/granmo/ (accessed on 14 June 2022)). Assuming a two-way ANOVA design (diet × sex), with an alpha risk of 0.05 and a beta risk < 0.2 (power ≥ 80%) in a two-sided test, a total of 7 animals per dietary group and sex was required to detect biologically relevant differences in lipidomic profiles.

### 2.3. Determination of FBG, OGTT, and FI

Animals were fasted for 6 h during the light phase before the assay and blood was collected from the saphenous vein to measure FI and FBG. To carry out OGTT, a solution of glucose was orally administered (2 g/kg BW in water), and saphenous blood was collected at 15, 30, 60, 90, and 120 min. Glycemia was evaluated using test strips and a Safe AQ pro I glucometer (Changsha Sinocare Inc., Changsha, Hunan, China). Concentrations of plasma insulin were determined with the Rat Insulin ELISA kit (Mercodia AB, Uppsala, Sweden; assay range 0.15–5.5 µg/L) following the manufacturer’s instructions. Homeostasis model assessment of insulin resistance (HOMA-IR) was calculated by the following formula: ([FI (µIU/mL) × FBG (mg/dL)]/405) [19,20].

### 2.4. Plasma Leptin and Adiponectin Quantification

The ProcartaPlex^TM^ Multiplex immunoassay (Thermo Fisher Scientific, Vienna, Austria) was used to quantify plasma leptin concentrations. A Luminex Instrument and ProcartaPlex Analyst Software v1.0 (MAGPIX^®^ analyzer, Luminex Corporation, Austin, TX, USA; assay range 17–17,325 pg/mL) at the Flow Cytometry Unit (FCU) of the Scientific and Technological Centers of the University of Barcelona (CCiT-UB) were used. Adiponectin concentrations in plasma were measured with the ADP/Acrp30 ELISA kit (Elabscience Biotechnology Inc., Houston, TX, USA; assay sensitivity 0.94 ng/mL). Data were analyzed using Multiskan Ascent v.2.6 software (Thermo Fisher Scientific, Vienna, Austria). All procedures followed the manufacturer’s protocols and were consistent with previously described methods [21,22].

### 2.5. Lipid Profiling Assessment

The semiquantitative lipidomics assay was conducted at Eurecat (Centre Tecnològic de Catalunya) using the package MetLC Lipidomics-II platform. A semiquantitative LC-MS-based assay for quantification of lipid species, including lysophosphocholines, phosphocholines, sphingomyelins, diacylglycerides, triacylglycerides, cholesteryl esters, and others, was applied.

Lipid extraction from plasma and fecal samples was performed using a liquid–liquid extraction method based on the Folch procedure [23]. Briefly, plasma (20 μL) or wet fecal samples (10 mg) were mixed with chloroform:methanol (2:1, *v*/*v*) in the presence of a commercial internal standard mixture (Lipidomic SPLASH^®^). Fecal samples were mechanically homogenized using a Bullet Blender^®^ (Next Advance, Inc., Troy, NY, USA) for 3 min. Samples were incubated at −20 °C to enhance lipid precipitation, followed by phase separation induced by the addition of saline solution (0.9% NaCl). Following centrifugation, the organic phase containing lipids was collected, evaporated to dryness, and reconstituted in methanol:methyl-tert-butyl ether (9:1, *v*/*v*) prior to instrumental analysis.

Lipidomic profiling was performed using an ultra-high-performance liquid chromatography system coupled to quadrupole time-of-flight mass spectrometry (UHPLC-qTOF; Agilent 6550, Agilent Technologies, Santa Clara, CA, USA) operated in positive electrospray ionization mode.

Chromatographic separation was achieved on a C18 reversed-phase column (Kinetex EVO C18, 2.6 μm, 2.1 × 100 mm; Phenomenex) using a ternary mobile phase composed of water, methanol, and 2-propanol supplemented with 10 mM ammonium formate and 0.1% formic acid.

Lipid species for semi-targeted analysis were annotated using MassHunter Lipid Annotator software v1.0 (Agilent Technologies, Santa Clara, CA, USA) following the workflow described by Koelmel et al. (2020) [24]. To increase MS/MS coverage and facilitate the annotation of low-abundance lipid species, pooled samples were analyzed using a data-dependent iterative MS/MS acquisition strategy, in which precursor ions fragmented in previous injections were excluded from subsequent MS/MS events. Lipid annotation was based on accurate mass, isotopic pattern, and tandem mass spectral information, when available, and was further supported by chromatographic behavior of authentic standards representative of each lipid family and previously reported literature data. Additional comparison with the METLIN Personal Compound Database and Library (PCDL; Agilent Technologies) was performed when appropriate.

A pooled quality-control (QC) sample was analyzed every 10 study samples throughout the analytical sequence, resulting in 8 QC injections for each matrix (plasma and feces). QC samples were used to monitor analytical performance and signal stability throughout the analytical sequence. The relative standard deviation (RSD, %) across QC injections was calculated for each detected lipid species as a measure of analytical variability. Median RSD values and ranges by lipid class are reported in Supplementary Table S2. No batch correction was applied, as samples from each matrix were analyzed within a single analytical batch. In addition, no post-acquisition QC-based signal correction was applied, according to the analytical laboratory’s QC criteria. This QC-based correction should be distinguished from the internal-standard normalization used for semiquantification, which is described above.

Results were obtained as µmol of lipid species per liter of plasma or as nmol of lipid species per gram of wet feces. Lipid species were annotated according to the total number of carbon atoms and double bonds in the combined fatty acyl chains. For sphingolipids in plasma, including ceramides and sphingomyelins, lipid species were reported according to the sphingoid base and the *N*-acyl FA moiety, where the sphingoid base is indicated first followed by the FA chain.

Lipid species were semiquantified using class-specific external calibration or internal-standard-based approaches. For lipid classes with authentic standards available, calibration curves were generated using LPC 18:0, PC 32:0, SM 34:1, TG 52:3, and ChoE 16:0 from Avanti Polar Lipids (Alabaster, AL, USA). Analyte peak areas were normalized to the corresponding isotope-labeled internal standards. For lipid classes without external standards (LPE, MG, DG, PE, and PE-O), concentrations were estimated from the analyte-to-internal-standard peak-area ratio using the known concentration of the corresponding isotope-labeled internal standard present in the SPLASH Lipidomix^®^ mixture (Avanti Polar Lipids). Results were reported as internal-standard equivalents, assuming equivalent MS response between the analyte and its assigned internal standard. The internal standards used for plasma and fecal lipidomics are detailed in Supplementary Tables S3 and S4, respectively.

ACar were semiquantified separately using authentic unlabeled standards from Cambridge Isotope Laboratories (Carnitine/Acylcarnitine Standards Mix B, Item No. NSK-B-US-1). Calibration curves were generated using the corresponding authentic standard when available or the most structurally similar ACar standard for analytes without an exact match.

### 2.6. Statistical Analysis

No inclusion or exclusion criteria were established, and no animals or data points were excluded from the analyses. Blinding was not performed during experimental conduction and data analysis. Statistical analyses of physiological, metabolic, and lipid class/subclass data were performed using IBM SPSS Statistics (version 30.0; IBM Corp., Chicago, IL, USA) and RStudio (version 2026.01.0; Posit Software, PBC) with R version 4.3.2 (R Core Team, R Foundation for Statistical Computing, Vienna, Austria) [25]. Normality and homoscedasticity were assessed using the Shapiro–Wilk’s and Levene’s test, respectively. Once these conditions were confirmed, a two-way ANOVA test with diet and sex as fixed factors was applied. When a significant diet × sex interaction was detected, simple effects were examined by pairwise comparisons of the estimated marginal means. For variables that did not meet the assumptions of parametric analysis, a non-parametric aligned rank transform ANOVA (ART-ANOVA) was performed using the ARTool package in R. When a significant diet × sex interaction was observed, simple effects were evaluated using the emmeans package based on the aligned rank transformed model. Data are expressed as mean ± standard error of the mean (SEM), and significant differences were considered when *p* < 0.05.

For multivariate and individual lipid species analysis, data were processed using MetaboAnalyst 6.0 (MetaboAnalyst). Prior to statistical analysis, data were preprocessed using sample normalization by median, logarithmic (base 10) transformation, and data scaling (mean-centered and divided by the standard deviation of each variable). Principal component analysis (PCA) was used to search for clusters of similarities between samples in terms of lipidomic composition. To statistically assess group separation in the 2D PCA plots, permutational multivariate analysis of variance (PERMANOVA) was conducted based on Euclidean distance matrices calculated from the PC1 and PC2 scores, with 999 permutations. For individual lipid species, two-way ANOVA was used to assess the main effects of diet, sex, and their interaction. Pairwise *t*-tests were used for the corresponding within-sex dietary comparisons. Benjamini–Hochberg false discovery rate (FDR) correction was applied to the individual lipid species analyses to account for multiple comparisons, and FDR-adjusted *p*-values < 0.05 were considered statistically significant. Volcano plots and Venn diagrams were generated to visualize significantly altered lipid species and overlapping features between comparisons. Data are expressed as mean ± SEM.

Spearman correlation analyses were performed separately within each dietary group to assess associations between lipid species and physiological and metabolic variables. Multiple testing was controlled using the FDR procedure, and only correlations with FDR-adjusted *p* < 0.05 were considered significant. Correlation matrices and heatmaps were generated using the Hmisc v5.2.3 and pheatmap v1.0.12 R packages, respectively.

## 3. Results

### 3.1. Morphometric and Metabolic Characterization of the Experimental Model

HFD feeding led to an increase in BW gain throughout the 12 weeks of study, as well as in BMI in both females and males (Table 1). However, HFD did not alter the Lee index and the WC. As expected, sex-related differences in morphometric variables were observed regardless of diet, with male rats showing greater BW gain, BMI, Lee index, and WC compared to females.

Diet-induced changes were observed in the glycemic profile, although no sex-dependent effect was found. In the OGTT, animals receiving the HFD displayed higher area under the curve (AUC) compared to REF animals, reflecting impaired glucose tolerance (Table 1). A significant diet × sex interaction was observed for FBG levels, with HFD females showing significantly lower FBG levels compared to the REF group, and HFD males showing higher FBG levels compared to REF, although this difference did not reach statistical significance. No differences were observed in the levels of FI and the HOMA-IR. With respect to adipokine levels in plasma, no differences were found in the levels of leptin and adiponectin; however, HFD males displayed an increased leptin/adiponectin ratio compared to REF males (Table 1). Moreover, males displayed higher levels of leptin and lower levels of adiponectin than females.

### 3.2. Multivariate Analysis of Plasma and Fecal Lipidomic Profiles

A total of 170 lipid species were detected in plasma and 126 in feces, encompassing a range of lipid categories including fatty acyls, glycerolipids, glycerophospholipids, sphingolipids, and sterols (Table S5).

**Figure 1:**
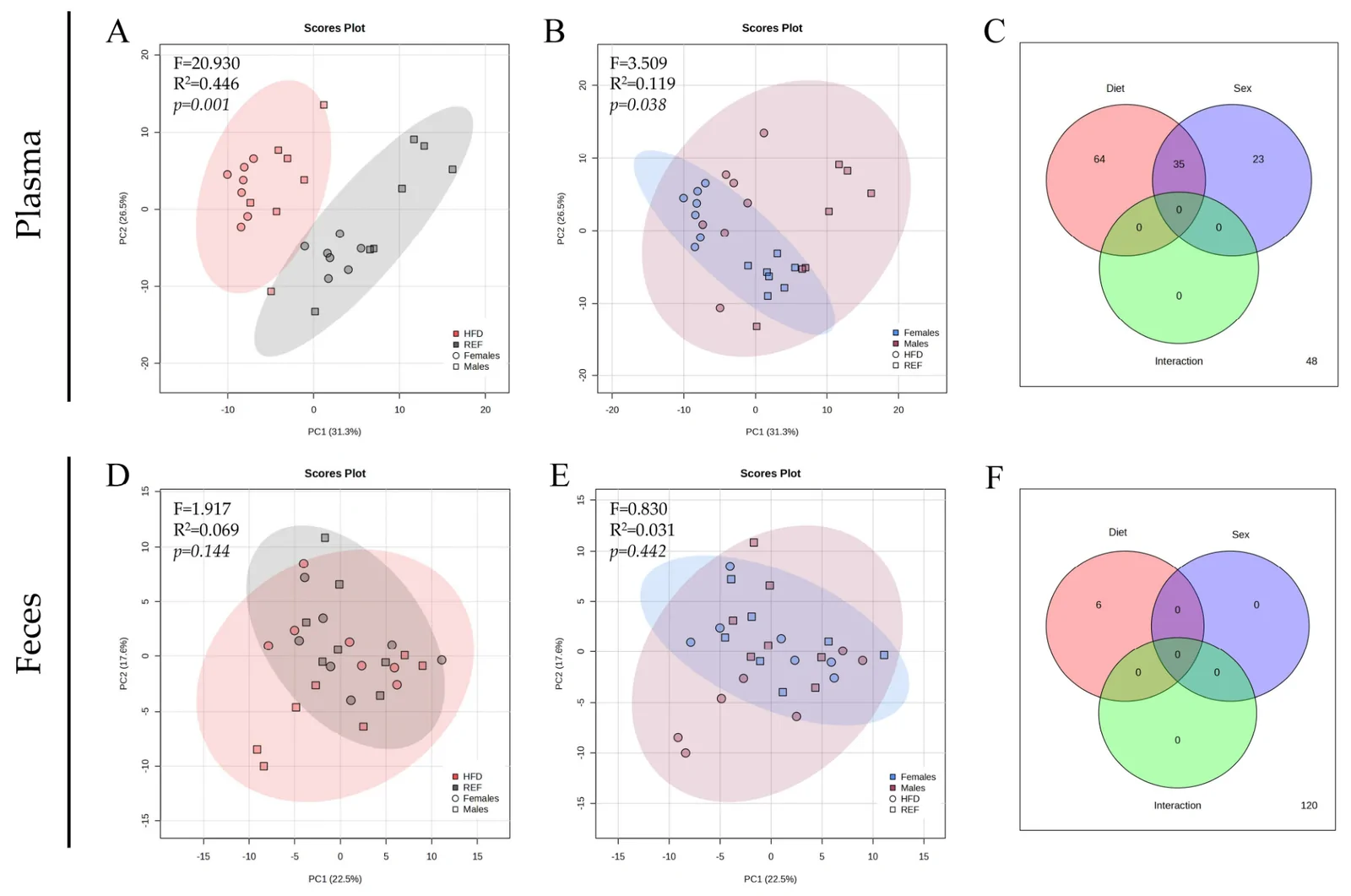
Matrix-dependent effects of HFD and sex on plasma and fecal lipidomic profiles. PCA score plot of the plasma lipidomic profile based on (**A**) diet-associated separation and (**B**) sex-associated separation. (**C**) Venn diagram summarizing the number of plasma lipid species significantly modulated by diet, sex, and their interaction, as determined by a two-way ANOVA of transformed data with FDR correction (*p*-adj < 0.05). PCA score plot of the fecal lipidomic profile based on (**D**) diet-associated separation and (**E**) sex-associated separation. (**F**) Venn diagram summarizing the number of fecal lipid species significantly modulated by diet, sex, and their interaction, as determined by a two-way ANOVA of transformed data with FDR correction (*p*-adj < 0.05). Statistical significance (*p* < 0.05) of group separation was assessed using PERMANOVA based on Euclidean distances computed from the PCA scores displayed (PC1 and PC2; *n* = 7/group and sex).

To investigate the overall impact of HFD exposure and sex-dependent differences on lipidomic composition, PCA was applied to both plasma and fecal datasets to assess sample clustering and variation (Figure 1). In plasma, PCA revealed a clear differential clustering of samples according to dietary intervention, indicating a significant restructuring of the circulating lipidome upon HFD feeding further confirmed by PERMANOVA (*p* = 0.001; Figure 1A). When samples were colored according to sex, a partial segregation between females and males was also observed, with PERMANOVA further demonstrating that sex significantly influenced plasma lipid composition (*p* = 0.038; Figure 1B). In contrast, PCA of the fecal lipidome did not show distinct clustering according to HFD exposure or sex, reflecting a largely overlapping distribution of samples across groups (Figure 1D,E). PERMANOVA supported this observation, revealing no significant effect of HFD feeding (*p* = 0.144) or sex (*p* = 0.442) on the global fecal lipidomic profile.

The distribution of significantly regulated species was visualized using Venn diagrams, allowing identification of lipids predominantly influenced by diet, sex, or their interaction (Table S6). In plasma, a substantial number of lipid species were significantly altered by HFD exposure (n = 99), while a smaller subset exhibited sex-dependent differences (n = 58). No significant diet × sex interaction was identified at the species level (Figure 1C). In feces, only six lipid species were significantly altered in response to HFD exposure, while no species were significantly associated with sex or diet × sex interaction, consistent with the absence of global separation observed in PCA (Figure 1F).

### 3.3. Total, Class, and Subclass-Specific Lipid Levels in Plasma and Feces

The identified lipid species in plasma included ceramides (Cer), sphingomyelins (SM), cholesteryl esters (ChoE), phosphatidylcholines (PC), phosphatidylethanolamines (PE), lysophosphatidylcholines (LPC), lysophosphatidylethanolamines (LPE), monoacylglycerols (MG), diacylglycerols (DG), and triacylglycerols (TG; Table S5). In feces, a partially overlapping set of lipid classes was identified, including these same classes/subclasses with the addition of ACar and phosphatidylserines (PS), and no identification of MG.

**Figure 2:**
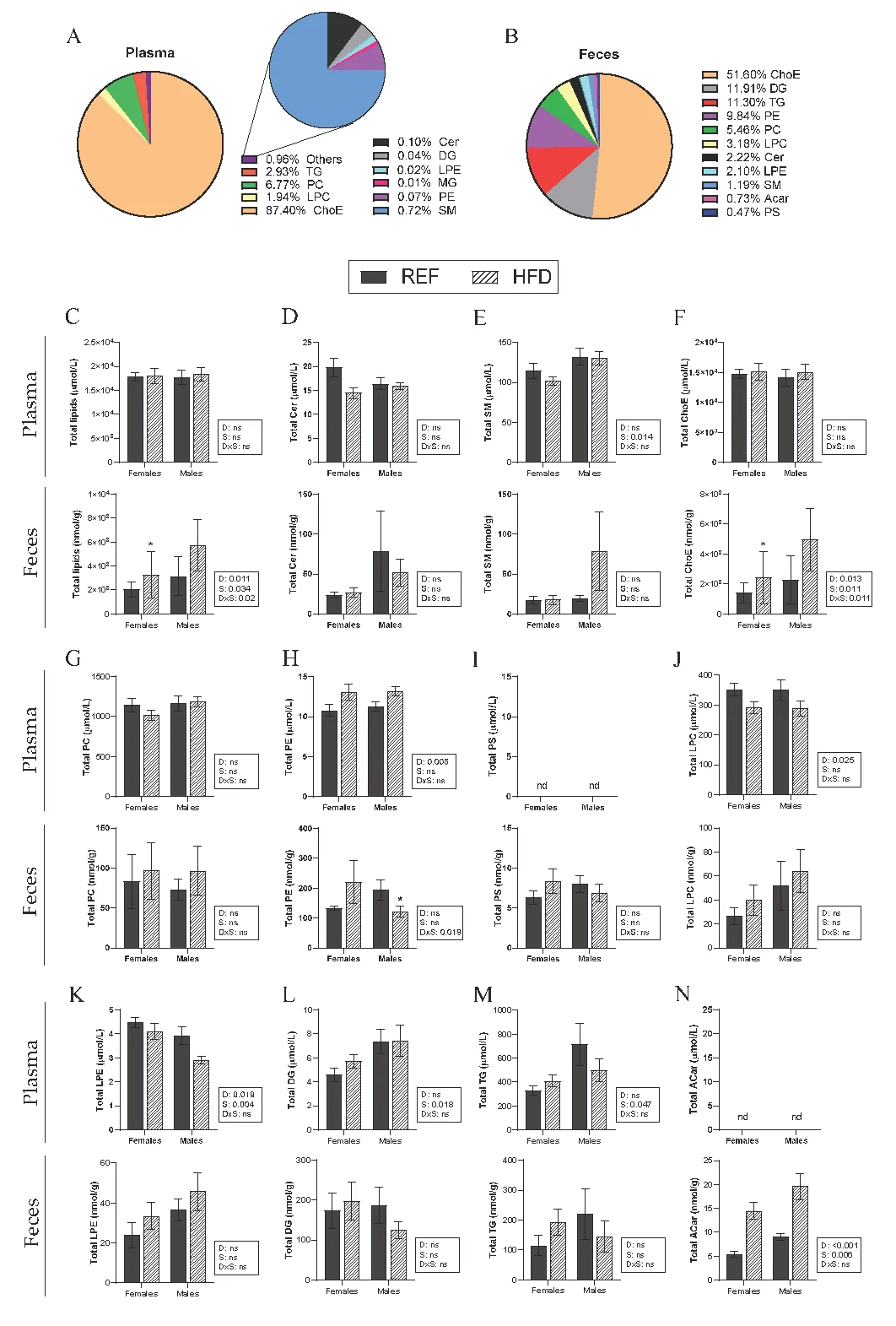
Relative abundances and total levels of lipid classes and subclasses in the plasma and feces of rats undergoing dietary intervention. Relative abundances of lipid classes and subclasses in (**A**) plasma and (**B**) feces. Plasma and fecal concentration of (**C**) total lipids, (**D**) total ceramides (Cer), (**E**) total sphingomyelins (SM), (**F**) total cholesteryl esters (ChoE), (**G**) total phosphatidylcholines (PC), (**H**) total phosphatidylethanolamines (PE), (**I**) total phosphatidylserines (PS), (**J**) total lysophosphatidylcholines (LPC), (**K**) total lysophosphatidylethanolamines (LPE), (**L**) total diacylglycerols (DG), (**M**) total triacylglycerols (TG), and (**N**) total acylcarnitines (ACar). Pie charts (**A**,**B**) represent the average relative contribution of each lipid class/subclass considering all animals included in the study, irrespective of sex or experimental group. Total plasma and fecal lipid content was calculated as the sum of the quantified lipid species included in the lipidomic analysis for each compartment. Data are expressed as mean ± SEM. Statistical significance: *p*-values for the main effects of diet, sex, and their interaction were obtained by two-way ANOVA or ART-ANOVA, as appropriate, and are indicated in the figure (**C**–**N**) (*n* = 7/group and sex). When a significant diet × sex interaction was detected (*p* < 0.05), simple effects were analyzed: * *p* < 0.05 vs. the corresponding REF group. D, diet; S, sex; D × S, diet × sex interaction; ns, no statistical significance detected; nd, non-detected.

Although plasma and feces shared the same major lipid classes and subclasses, their relative distributions differed (Figure 2A,B). In plasma, ChoE were the most abundant lipids (more than 85%), followed by PC (about 7%), TG (about 3%), and LPC (about 2%; Figure 2A). The fecal lipidome was also dominated by ChoE (more than 50%) but showed a distinct class distribution, with DG, TG, and PE (Figure 2B) representing the next most abundant classes (with 10–12% relative abundances). All other lipid classes contributed with smaller proportions than 6% to the overall fecal lipid composition.

Clear differences emerged between plasma and fecal matrices when total lipid levels were considered (Figure 2C). In plasma, no significant differences in total lipid content were detected between REF and HFD groups or between sexes. In contrast, total fecal lipid levels were significantly influenced by diet and sex interaction, with HFD females displaying higher overall fecal lipid levels compared with REF animals.

Class/subclass-level analysis further highlighted distinct patterns in plasma and fecal lipidomes. Within sphingolipids, total Cer levels were not significantly altered in either plasma or feces (Figure 2D). However, total SM levels in plasma were significantly influenced by sex, with males displaying higher levels than females, whereas no diet effect was detected. In contrast, total SM levels in feces did not differ between experimental groups (Figure 2E).

ChoE concentrations also showed matrix-specific responses (Figure 2F). In plasma, total ChoE levels were not significantly affected by diet or sex. However, in feces, ChoE concentrations were significantly influenced by diet and sex interaction, with HFD females increasing ChoE levels.

Among glycerophospholipids, total PC levels were not significantly altered by diet or sex in either matrix (Figure 2G). On the other hand, in plasma, total PE levels were significantly increased following HFD exposure in both sexes, whereas in feces PE levels exhibited a significant diet × sex interaction, decreasing in HFD-fed males (Figure 2H). In feces, PS concentrations did not show alterations due to HFD or sex (Figure 2I). Concerning LPC levels, they were significantly reduced in plasma under HFD conditions, while fecal LPC concentrations remained unchanged (Figure 2J). Plasma LPE levels were reduced by HFD and by male sex, whereas in feces, they were not influenced by either diet or sex (Figure 2K).

Total DG and TG plasma levels were higher in males than females independently of diet. In contrast, neither fecal DG nor TG contents were significantly affected by HFD or sex (Figure 2L,M).

Finally, a lipid subclass uniquely detected in feces, ACar, showed a marked increased concentration due to diet and in male animals (Figure 2N).

### 3.4. Individual Plasma and Fecal Lipid Species: Diet- and Sex-Based Comparisons

**Figure 3:**
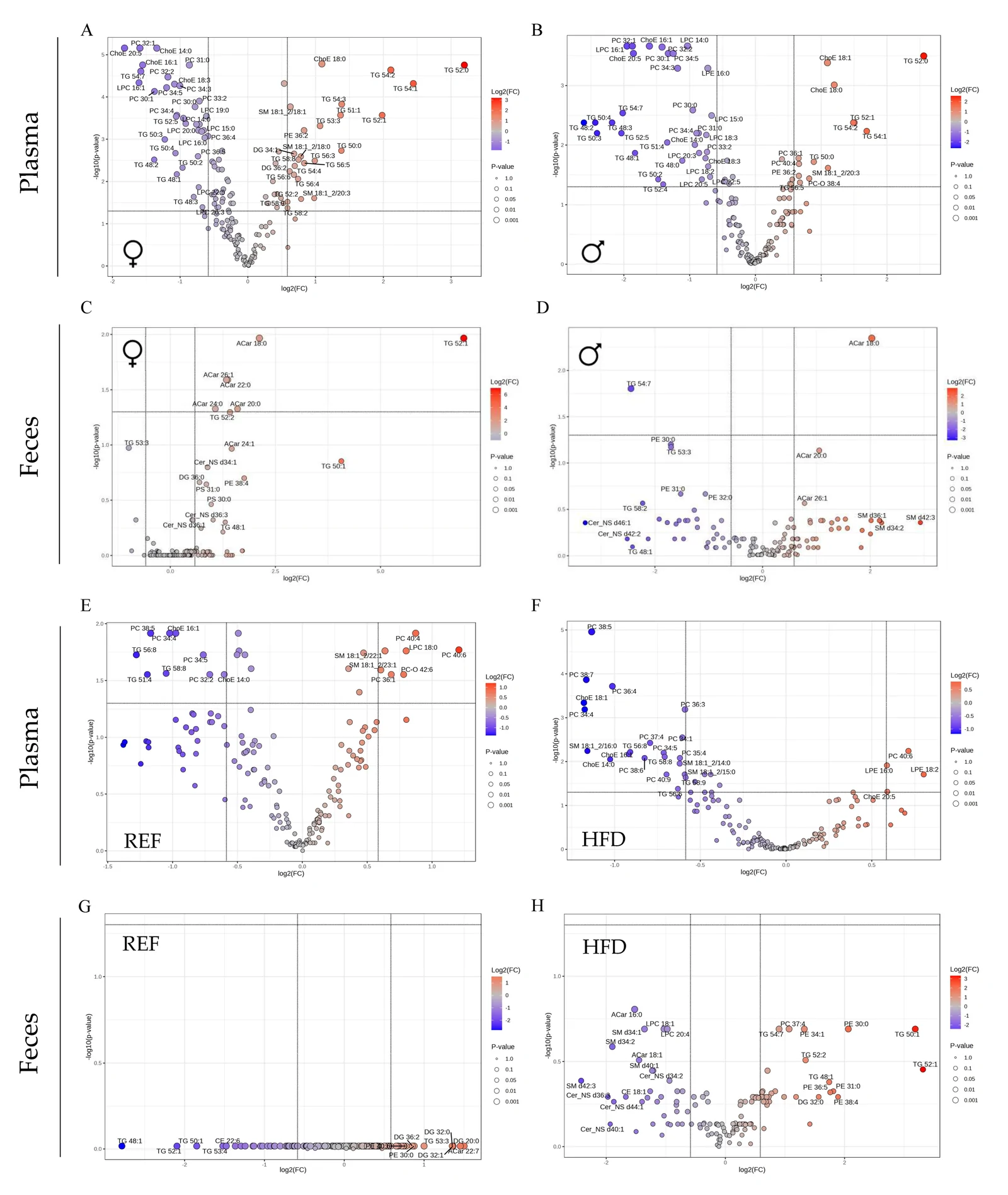
Diet- and sex-dependent alterations in plasma and fecal lipid species. Volcano plot representation of altered lipid metabolites of (**A**) HFD vs. REF groups within females in plasma, (**B**) HFD vs. REF groups within males in plasma, (**C**) HFD vs. REF groups within females in feces, and (**D**) HFD vs. REF groups within males in feces, and females vs. males within the (**E**) REF group and (**F**) HFD group in plasma, and females vs. males within the (**G**) REF group and (**H**) HFD group in feces. Volcano plots display log_2_ of the FC versus −log_10_ of the adjusted *p*-value, highlighting lipid species significantly altered between groups when the dots are located over the horizontal line (*p*-adj < 0.05). Vertical lines represent the 1.5-FC boundary used to identify lipid species with substantial variation between groups. The red dots indicate lipids that are increased, and blue dots represent those decreased in HFD (**A**–**D**) and females (**E**–**H**) (*n* = 7/group and sex).

To further characterize lipid alterations induced by HFD and/or sex at the species level, volcano plot analyses were performed in both plasma and fecal lipidomes (Figure 3). Significantly altered lipid species were defined by an adjusted *p*-value < 0.05 and fold changes (FC) ≥ 1.5. A complete list of significantly altered plasma lipid species is displayed in Table S7.

Diet-induced changes showed a markedly different pattern between the two matrices. In plasma, HFD exposure induced substantial remodeling of the lipidome in both sexes (Figure 3A,B). When comparing females under HFD vs. REF, a substantial number of lipid species were significantly altered in both directions, indicating extensive lipidomic remodeling rather than selective changes affecting only a limited set of lipids. A comparable pattern was observed in males, with a large number of lipid species significantly regulated by HFD exposure. In contrast, diet-induced remodeling of the fecal lipidome at the species level was limited (Figure 3C,D). Only a small number of lipid species showed significant alterations in response to HFD in both sexes. In females, a subset of ACar species and TG 52:1 increased significantly, whereas in males only ACar 18:1 increased and TG 54:7 decreased following HFD exposure.

Sex-associated differences were comparatively modest both in plasma and fecal compartments. Under the REF diet, comparison between females and males revealed a limited number of significantly regulated lipid species in plasma (Figure 3E). Similarly, under HFD conditions, sex-related differences persisted and included some species already differing under REF conditions; however, these alterations remained confined to a discrete set of lipid species and did not indicate extensive sex-dependent remodeling of the plasma lipidome (Figure 3F). With regard to the fecal lipidome, no statistically significant sex-related differences at the species level were detected under either REF or HFD conditions (Figure 3G,H).

### 3.5. Plasma Lipidomics

To further investigate diet- and sex-associated changes in plasma, each lipid category was analyzed separately.

#### 3.5.1. Plasma Sphingolipids

**Figure 4:**
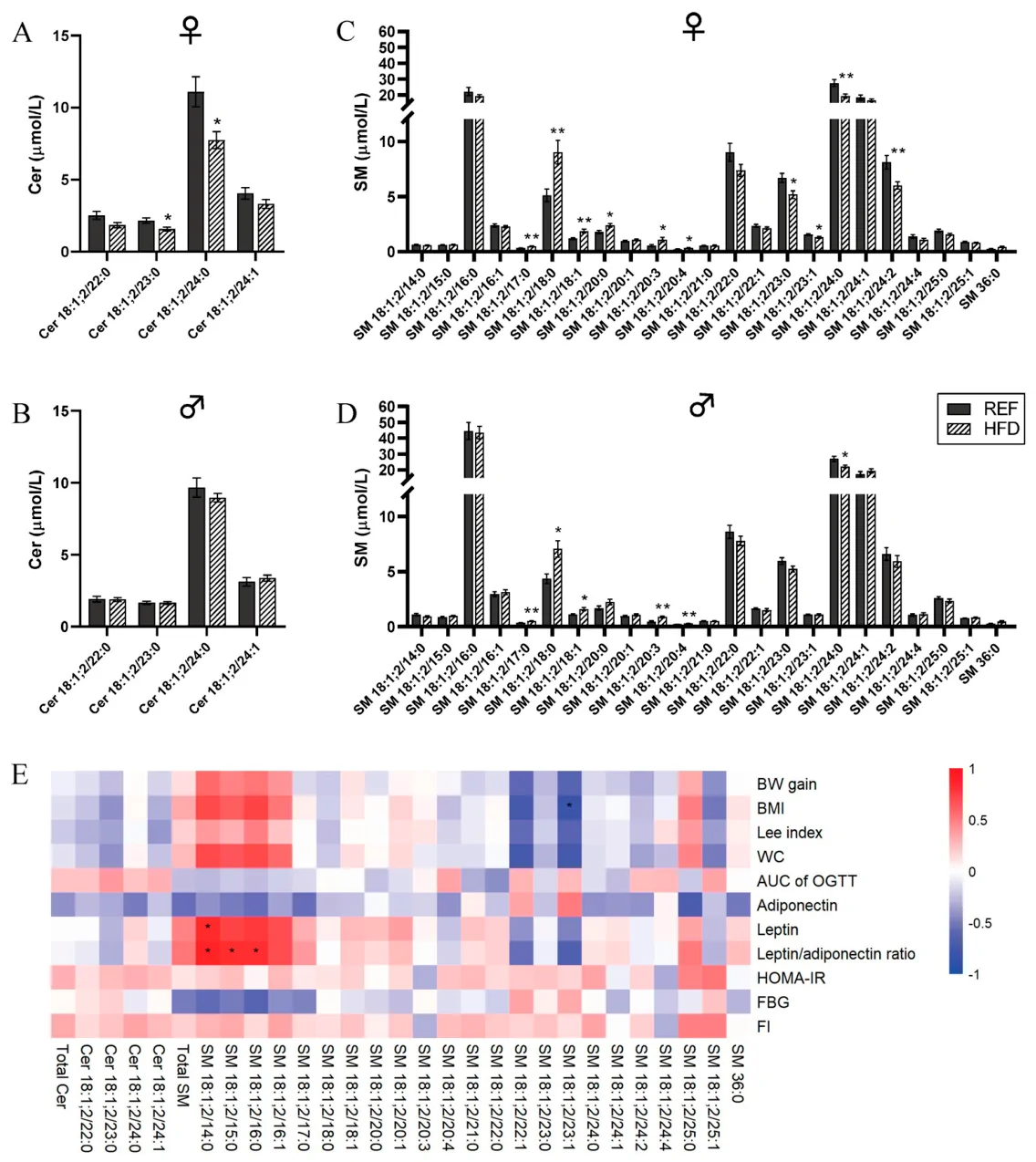
Plasma levels of ceramides (Cer) and sphingomyelins (SM) species. Plasma concentration detected in female rats showing (**A**) Cer species and (**C**) SM species and in male rats for (**B**) Cer species and (**D**) SM species, respectively. Data are expressed as mean ± SEM. Statistical significance was assessed by *t*-test of transformed data with FDR correction (* *p*-adj < 0.05, ** *p*-adj < 0.01; *n* = 7/group and sex). (**E**) Heatmap showing correlations between plasma Cer and SM species with physiological and metabolic variables associated with obesity in the HFD group (n = 14). Spearman correlation coefficients are represented by color, with blue indicating negative correlations and red indicating positive correlations. Statistically significant correlations after FDR correction are marked with asterisks: * *p*-adj < 0.05. BW gain, body weight gain; BMI, body mass index; WC, waist circumference; AUC OGTT, area under the curve of the oral glucose tolerance test; HOMA-IR, homeostatic model assessment of insulin resistance; FBG, fasting blood glucose; FI, fasting insulin.

Among the sphingolipids, four Cer species were detected (Figure 4A,B). Under HFD exposure, lower levels of two of the saturated Cer species (Cer 18:1;2/23:0 and Cer 18:1;2/24:0) were detected in females, whereas no changes were observed in males.

Among the SM, a total of 23 species were detected (Figure 4C,D). Under HFD, females produced higher levels of six species (SM 18:1;2/17:0, SM 18:1;2/18:0, SM 18:1;2/18:1, SM 18:1;2/20:0, SM 18:1;2/20:3, and SM 18:1;2/20:4) and lower concentrations of four species (SM 18:1;2/23:0, SM 18:1;2/23:1, SM 18:1;2/24:0, and SM 18:1;2/24:2). In males, the same SM species were also found in higher concentrations, except for SM 18:1;2/20:0, and only SM 18:1;2/24:0 content was significantly decreased in the males.

Correlation analyses were performed to assess the relationship between sphingolipid species and physiological and metabolic variables associated with obesity. Within the REF group, positive associations were observed between SM 18:1;2/14:0, SM 18:1;2/15:0, and SM 18:1;2/16:0 and the leptin-to-adiponectin ratio. In addition, SM 18:1;2/14:0 was positively associated with leptin, whereas SM 18:1;2/23:1 showed a negative association with BMI (Figure 4E; Table S8). In contrast, no significant associations between sphingolipid species and the assessed physiological or metabolic variables were detected within the HFD group after FDR correction (Figure S1).

#### 3.5.2. Plasma Cholesteryl Esters

**Figure 5:**
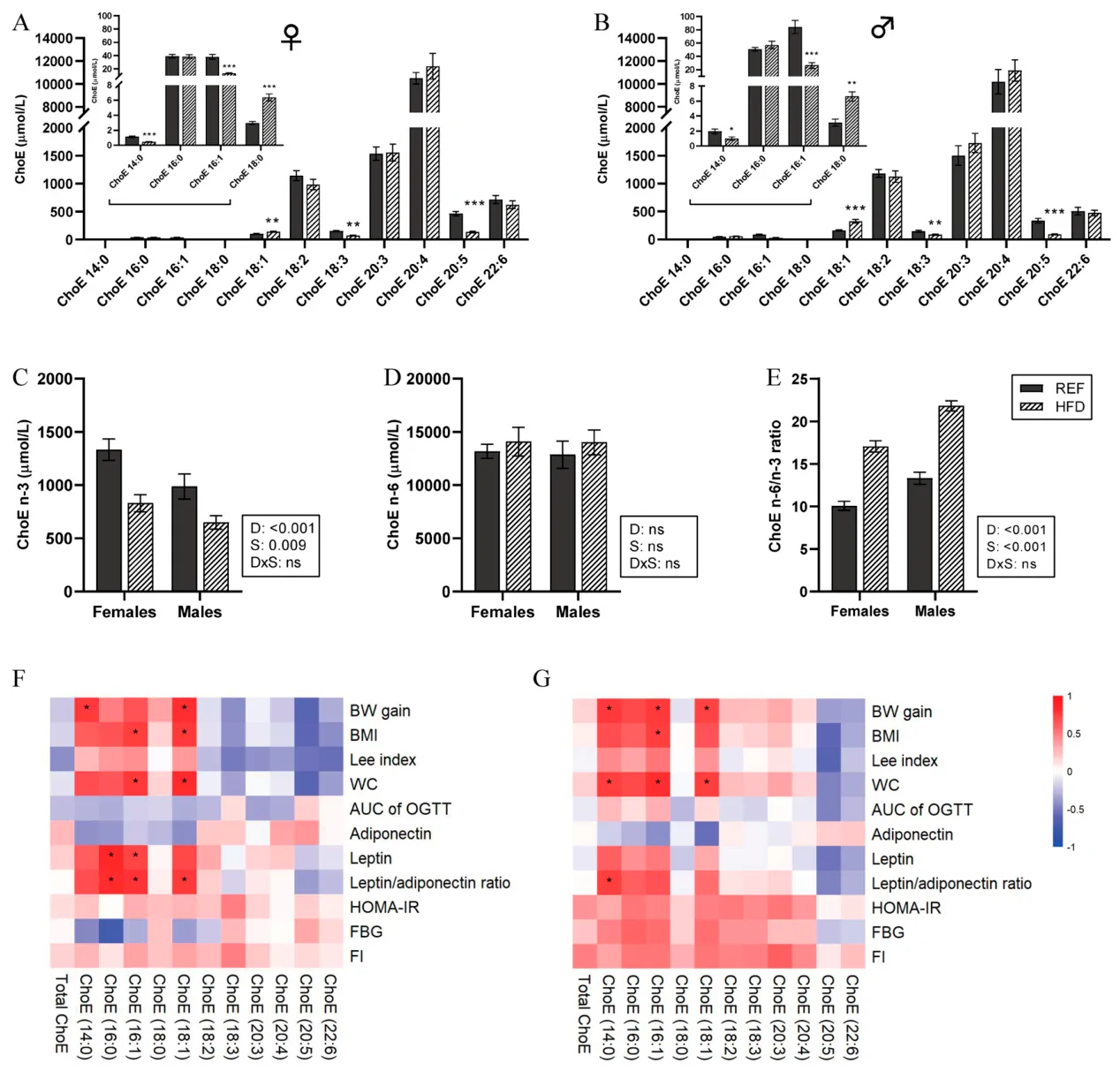
Plasma levels of cholesteryl ester (ChoE) species and *n*-6/*n*-3 ChoE composition. Plasma concentration of (**A**) detected ChoE species in female rats and (**B**) male rats, (**C**) ChoE *n*-3, and (**D**) ChoE *n*-6. (**E**) ChoE *n*-6/*n*-3 ratio. Data are expressed as mean ± SEM. Statistical significance was assessed by *t*-test of transformed data with FDR correction (* *p*-adj < 0.05, ** *p*-adj < 0.01, *** *p*-adj < 0.001) (**A**,**B**). *p*-values obtained by two-way ANOVA/ART-ANOVA for the variables diet, sex, and their interaction are indicated in (**C**–**E**) (*n* = 7/group and sex). Inset shows a magnified view of the low-abundance ChoE species. Heatmap showing correlations between plasma ChoE species with physiological and metabolic variables associated with obesity in the (**F**) REF group (*n* = 14) and (**G**) HFD group (*n* = 14). Spearman correlation coefficients are represented by color, with blue indicating negative correlations and red indicating positive correlations. Statistically significant correlations after FDR correction are marked with asterisks: * *p*-adj < 0.05. D, diet; S, sex; D × S, diet × sex interaction; ns, no statistical significance; BW gain, body weight gain; BMI, body mass index; WC, waist circumference; AUC OGTT, area under the curve of the oral glucose tolerance test; HOMA-IR, homeostatic model assessment of insulin resistance; FBG, fasting blood glucose; FI, fasting insulin. ChoE *n*-3 species: ChoE 18:3, ChoE 20:5, and ChoE 22:6; ChoE *n*-6 species: ChoE 18:2, ChoE 20:3, and ChoE 20:4.

A total of 11 ChoE species were detected in plasma samples (Figure 5A,B). Both females and males exhibited diet-induced changes in five ChoE species, with consistent directions of change across sexes. HFD led to reductions in the content of ChoE 14:0, ChoE 16:1, ChoE 18:3, and ChoE 20:5, and increased concentrations in ChoE 18:0 and ChoE 18:1.

To further explore the FA composition of these lipids, ChoE species containing polyunsaturated fatty acids (PUFAs) were classified as omega-3 (*n*-3) and omega-6 (*n*-6). ChoE *n*-3 levels were lower in males than females upon both REF and HFD feeding (Figure 5C). In contrast, ChoE *n*-6 levels did not show significant differences associated with either diet or sex (Figure 5D). Consistent with these findings, the ChoE *n*-6/*n*-3 ratio was higher in males and increased by HFD in both sexes (Figure 5E).

Correlation analyses revealed several positive associations between individual ChoE species and obesity-related physiological variables, with broadly similar patterns across dietary groups (Figure 5F,G; Table S8). ChoE 14:0 and ChoE 18:1 were positively associated with BW gain in REF and HFD animals, while ChoE 18:1 showed additionally positive associations in the latter. Similarly, ChoE 16:1 was positively associated with BMI in both dietary groups, whereas ChoE 18:1 was additionally associated with BMI in REF animals. Both ChoE 16:1 and ChoE 18:1 were positively associated with WC in REF and HFD animals, with the addition of ChoE 14:0 in HFD animals. This species was also positively associated with the leptin-to-adiponectin ratio in HFD animals. Within the REF group, ChoE 16:0 and ChoE 16:1 were positively associated with circulating leptin and the leptin/adiponectin ratio, while ChoE 18:1 was also positively associated with the latter.

#### 3.5.3. Plasma Glycerophospholipids

**Figure 6:**
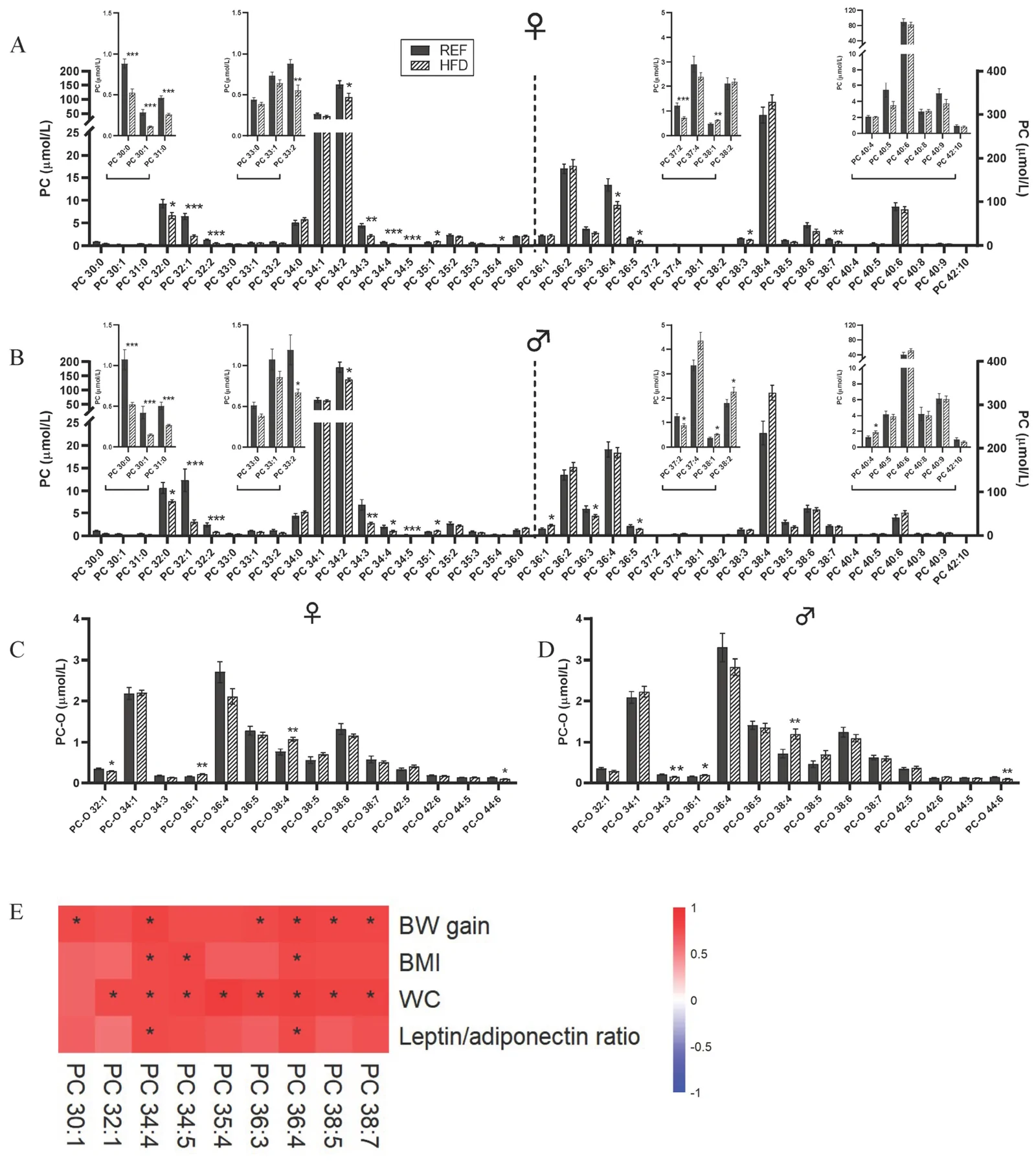
Plasma levels of phosphatidylcholine (PC) species. Plasma concentration of detected (**A**) diacyl PC species in female rats (**B**) and male rats, and (**C**) ether-linked phosphatidylcholine (PC-O) species in female rats (**D**) and male rats. Data are expressed as mean ± SEM. Statistical significance was assessed by *t*-test of transformed data with FDR correction (* *p*-adj < 0.05, ** *p*-adj < 0.01, *** *p*-adj < 0.001; *n* = 7/group and sex). Insets show a magnified view of the low-abundance species. (**E**) Heatmap showing correlations between plasma PC species with physiological and metabolic variables associated with obesity in the HFD group (*n* = 14). Spearman correlation coefficients are represented by color, with blue indicating negative correlations and red indicating positive correlations. Only species and variables that show significant correlations after FDR correction (* *p*-adj < 0.05) are displayed in the heatmap. BW gain, body weight gain; BMI, body mass index; WC, waist circumference.

PC represented the most abundant glycerophospholipid class detected in plasma, with a total of 54 species identified, spanning a wide range of concentrations (Figure 6). Several PC species showed consistent diet-associated alterations in both sexes. HFD reduced the plasma concentrations of PC 30:0, PC 30:1, PC 31:0, PC 32:0, PC 32:1, PC 32:2, PC 33:2, PC 34:2, PC 34:3, PC 34:4, PC 34:5, PC 36:5, and PC 37:2, and increased PC 35:1 levels in both females and males (Figure 6A,B). Other sex-specific differences observed include reductions in PC 36:4, PC 38:3, and PC 38:7 in males, while HFD males showed higher concentrations of PC 36:1, PC 38:2, and PC 40:4 than REF males, and reduced PC 36:3 concentration in HFD males.

Alterations in plasma levels of ether-linked phosphatidylcholines (PC-O) include a reduction in PC-O 32:1 in HFD females, PC-O 34:3 in HFD males, and PC-O 44:6 in both sexes. In contrast, levels of PC-O 36:1 and PC-O 38:4 were increased in HFD animals, regardless of sex (Figure 6C,D). Correlation analysis revealed no associations between PC species and the physiological and metabolic variables assessed in REF animals (Figure S2). In contrast, several positive associations were detected within the HFD group: PC 30:1, PC 34:4, PC 36:3, PC 36:4, PC 38:5, and PC 38:7 were associated with BW gain; PC 34:4, PC 34:5, and PC 36:4 with BMI; PC 34:4, PC 34:5, PC 36:4, PC 32:1, PC 35:4, PC 36:3, PC 38:5, and PC 38:7 with WC; PC 34:4 and PC 36:4 with the leptin/adiponectin ratio (Figure 6E; Table S8).

**Figure 7:**
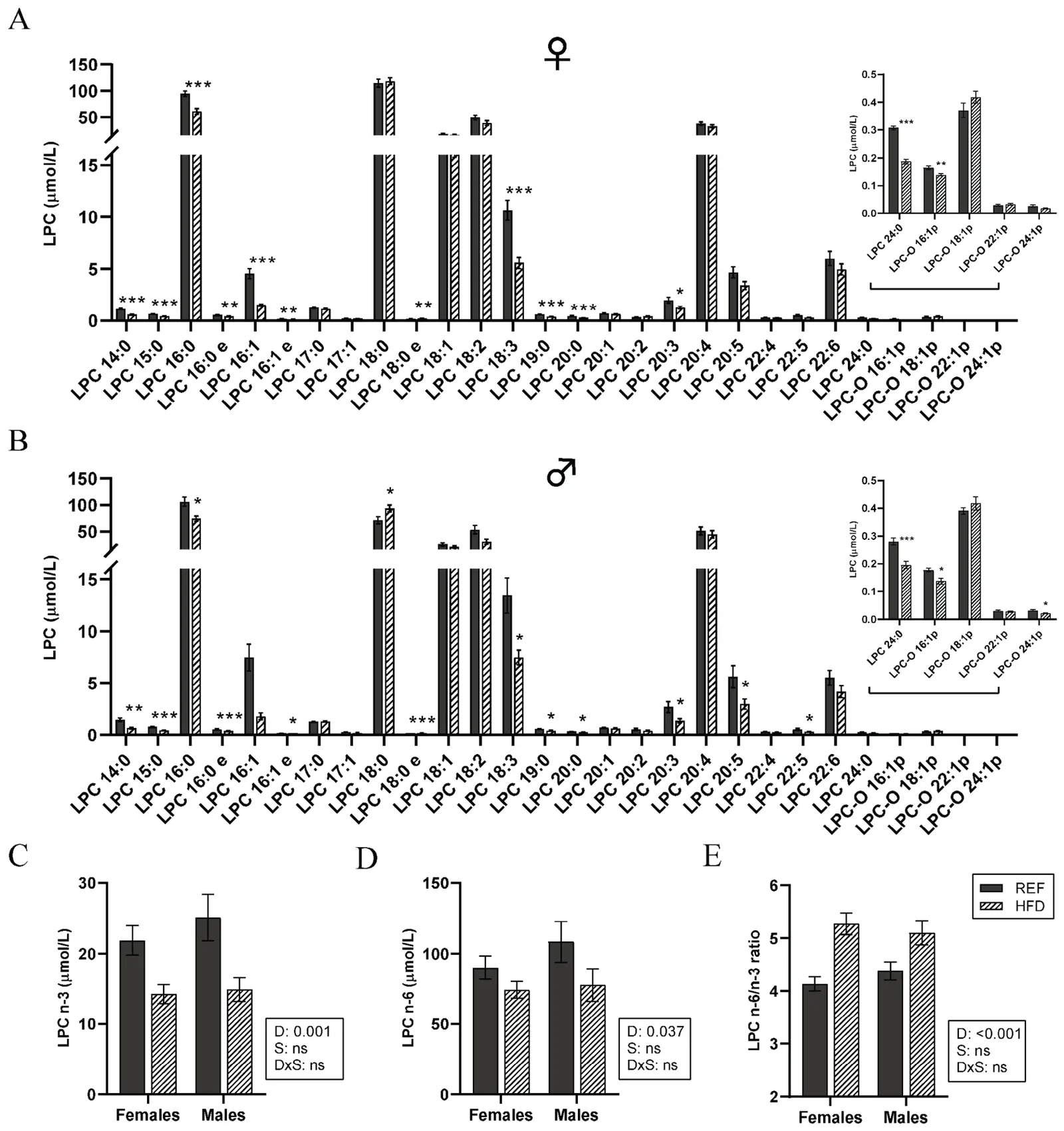
Plasma levels of lysophosphatidylcholine (LPC) species and *n*-6/*n*-3 LPC composition. Plasma concentration of LPC species in (**A**) female rats and (**B**) male rats, and ratios of (**C**) LPC *n*-3, (**D**) LPC *n*-6, and (**E**) LPC *n*-6/*n*-3. Data are expressed as mean ± SEM. Insets show a magnified view of the low-abundance species. Statistical significance was assessed by *t*-test of transformed data with FDR correction (* *p*-adj < 0.05, ** *p*-adj ≤ 0.01, and *** *p*-adj ≤ 0.001 vs. REF) (**A**,**B**). *p*-values obtained by two-way ANOVA/ART-ANOVA for the variables diet, sex, and their interaction are indicated in (**C**–**E**) (*n* = 7/group and sex). D, diet; S, sex; D × S, diet × sex interaction; ns, no statistical significance. LPC *n*-3 species: LPC 18:3, LPC 20:5, LPC 22:5, and LPC 22:6; LPC *n*-6 species: LPC 18:2, LPC 20:2, LPC 20:3, LPC 20:4, and LPC 22:4.

For the LPC, a total of 28 species were detected in plasma samples. In females, 14 LPC species (LPC 14:0, LPC 15:0, LPC 16:0, LPC 16:0e, LPC 16:1, LPC 16:1e, LPC 18:0e, LPC 18:3, LPC 19:0, LPC 20:0, LPC 20:3, LPC 24:0, and LPC-O 16:1p) were reduced in response to HFD (Figure 7A). In males, the same subset of species levels was altered except for LPC 16:1. Additional decreases in the concentrations of LPC 20:5, LPC 22:5, and LPC-O 24:1p and an increase of LPC 18:0 content were also observed in males (Figure 7B). To further examine potential differences in FA composition, LPC species were classified as omega-3 (*n*-3) and omega-6 (*n*-6; Figure 7C,D). HFD animals displayed lower levels of both LPC *n*-3 and LPC *n*-6 species than REF animals, resulting in a significantly increased LPC *n*-6/*n*-3 ratio in the HFD group in both sexes (Figure 7E). No significant correlations were observed between LPC species and the physiological and metabolic variables analyzed within each dietary group (Figure S3).

**Figure 8:**
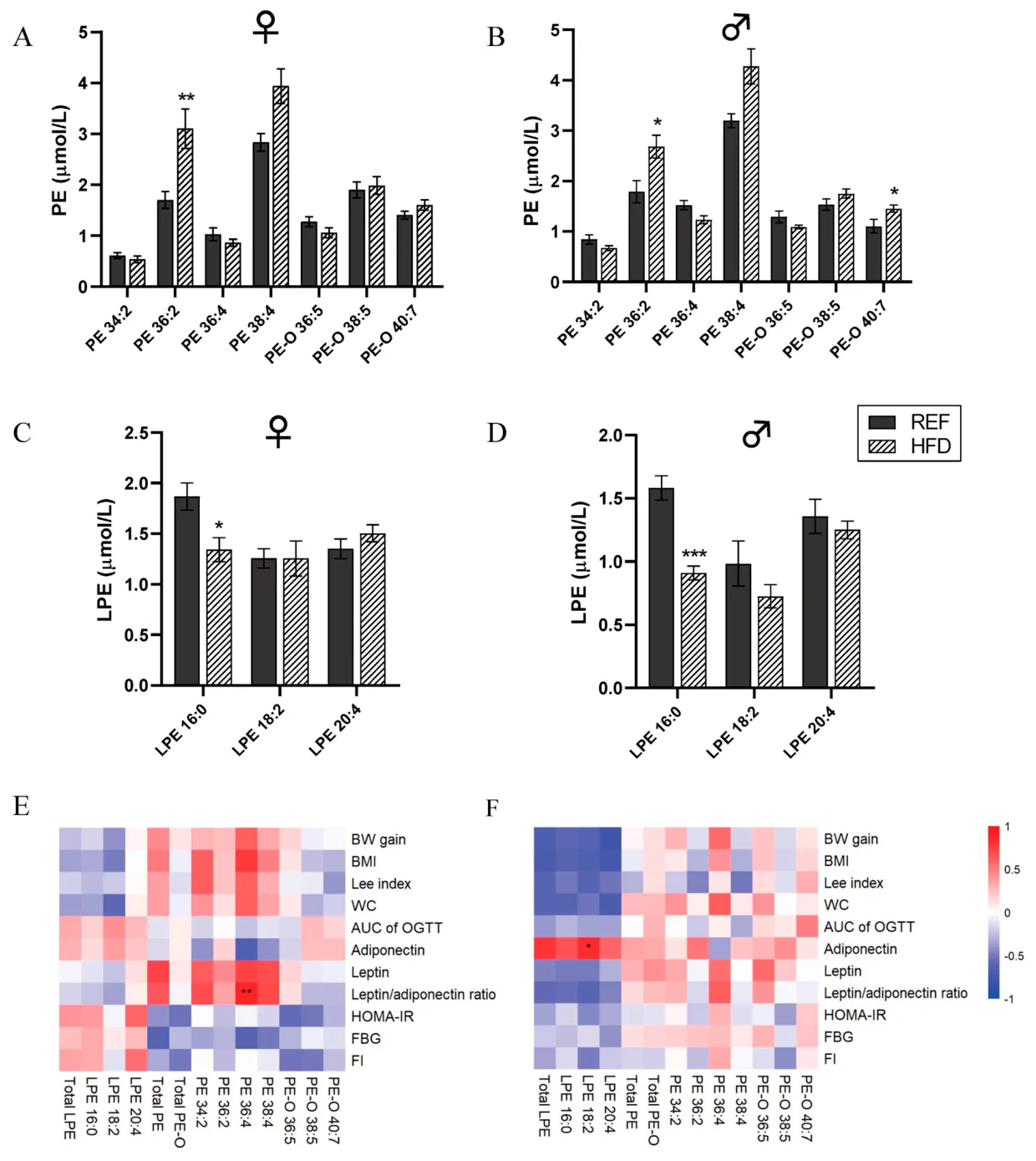
Plasma levels of phosphatidylethanolamines (PE) and lysophosphatidylethanolamines (LPE) species. Plasma concentration of PE species in (**A**) female rats and (**B**) male rats and LPE species in (**C**) female rats and (**D**) male rats. Data are expressed as mean ± SEM. Statistical significance was assessed by *t*-test of transformed data with FDR correction (* *p*-adj < 0.05, ** *p*-adj < 0.01, *** *p*-adj < 0.001; *n* = 7/group and sex). (**E**) Heatmap showing correlations between plasma LPE and PE species with physiological and metabolic variables associated with obesity in the (**E**) REF group (*n* = 14) and the (**F**) HFD group (*n* = 14). Spearman correlation coefficients are represented by color, with blue indicating negative correlations and red indicating positive correlations. Statistically significant correlations after FDR correction are marked with asterisks: * *p*-adj < 0.05. BW gain, body weight gain; BMI, body mass index; WC, waist circumference; AUC OGTT, area under the curve of the oral glucose tolerance test; HOMA-IR, homeostatic model assessment of insulin resistance; FBG, fasting blood glucose; FI, fasting insulin.

A total of seven species were detected as PEs; among these, HFD caused higher levels of PE 36:2 in both sexes and a higher concentration of PE-O 40:7 only in males (Figure 8A,B). For their lysophospholipid counterparts, three species were detected, but only LPE 16:0 levels were significantly reduced in HFD animals in both females and males (Figure 8C,D). Correlation analyses revealed that within the REF group the concentrations of PE 36:4 were directly associated with the leptin/adiponectin ratio (Figure 8E; Table S8). On the other hand, LPE 18:2 showed a positive correlation with adiponectin concentration within the HFD group.

#### 3.5.4. Plasma Glycerolipids

**Figure 9:**
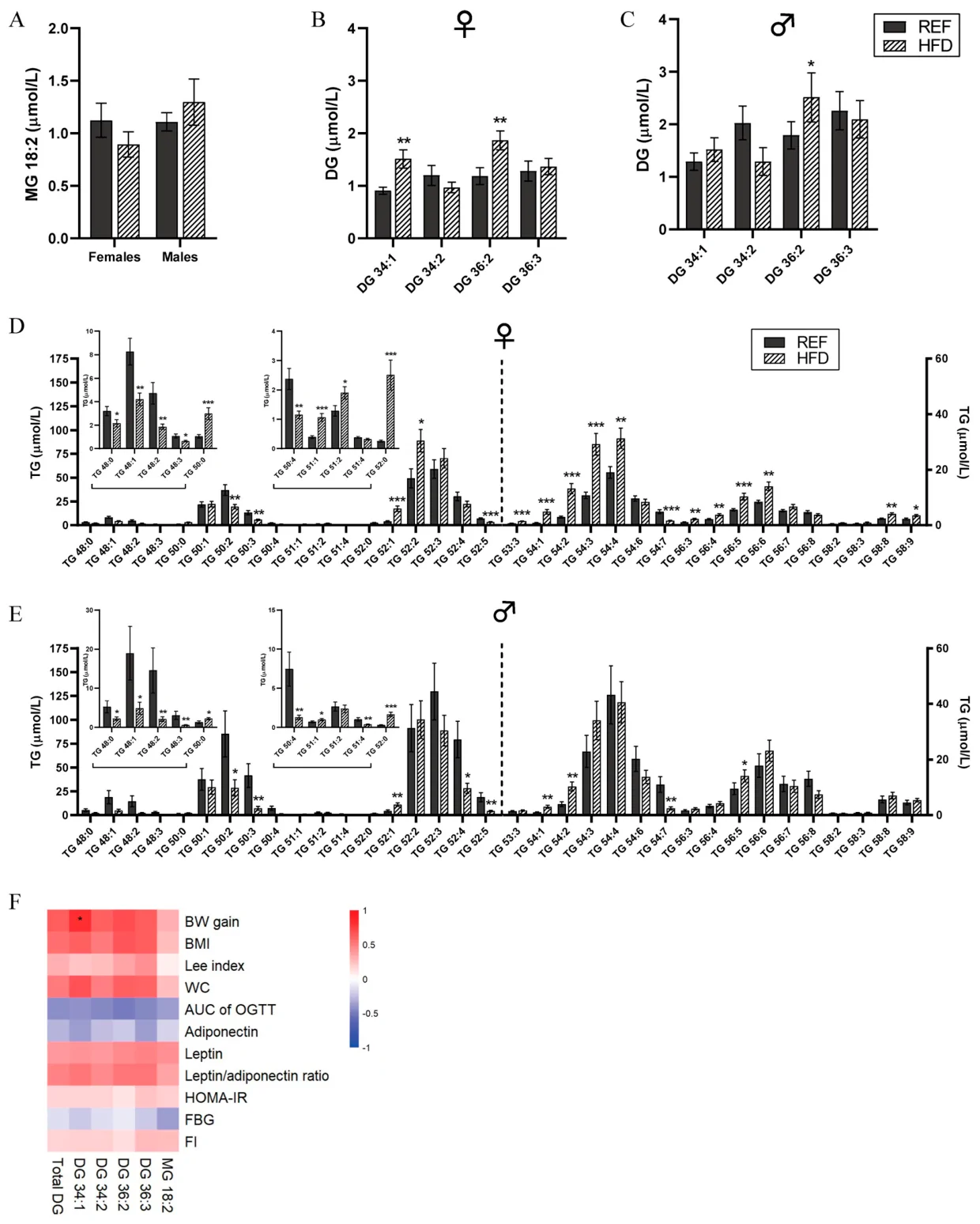
Plasma levels of diacylglycerols (DG) and triacylglycerols (TG) species. Plasma concentration of the (**A**) MG species in both female and male rats, DG species in (**B**) female and (**C**) male rats, and TG species in (**D**) female and (**E**) male rats. Data are expressed as mean ± SEM. Statistical significance was assessed by *t*-test of transformed data with FDR correction (* *p*-adj < 0.05, ** *p*-adj < 0.01, *** *p*-adj < 0.001; *n* = 7/group and sex). Insets show a magnified view of the low-abundance species. (**F**) Heatmap showing correlations between plasma MG and DG species with physiological and metabolic variables associated with obesity in the REF group (*n* = 14). Spearman correlation coefficients are represented by color, with blue indicating negative correlations and red indicating positive correlations. Statistically significant correlations after FDR correction are marked with asterisks: * *p*-adj < 0.05. BW gain, body weight gain; BMI, body mass index; WC, waist circumference; AUC OGTT, area under the curve of the oral glucose tolerance test; HOMA-IR, homeostatic model assessment of insulin resistance; FBG, fasting blood glucose; FI, fasting insulin.

Among MG, only MG 18:2 was detected and showed no differences between groups (Figure 9A). In contrast, among the DG species, four were associated with diet-associated changes (Figure 9B,C). DG 36:2 levels were higher in both HFD females and males compared to their REF counterparts. DG 34:1 concentration was higher in females consuming the HFD compared to REF diet.

Among the TG species, 35 were detected. Overall, HFD induced widespread remodeling of the TG profile in both sexes: 26 species in females and 19 species in males. The concentrations of the TG species TG 48:0, TG 48:1, TG 48:2, TG 48:3, TG 50:2, TG 50:3, TG 50:4, TG 52:5, and TG 54:7 were reduced in response to HFD in both sexes (Figure 9D,E). In males, concentrations of TG 51:4 and TG 52:4 were decreased. Conversely, the levels of several TG species, TG 50:0, TG 51:1, TG 52:0, TG 52:1, TG 54:1, TG 54:2, and TG 56:5, were elevated by HFD in both females and males. In HFD females, additional TG species that exhibited increased concentrations include TG 51:2, TG 52:2, TG 53:3, TG 54:3, TG 54:4, TG 56:3, TG 56:6, TG 58:8, and TG 58:9.

Within-diet correlation analyses revealed a positive association between DG 34:1 and BW gain in the REF group (Figure 9F; Table S8), while no significant associations were detected within the HFD group (Figure S4). Similarly, no significant correlations were identified between TG species and the assessed physiological and metabolic variables in either dietary group (Figure S5).

### 3.6. Fecal Lipidomics

Analysis of individual fecal lipid species (Figures S7–S10) confirmed the limited impact of HFD on the fecal lipidome. Most significant alterations were confined to the ACar subclass, except for the increase in the concentration of TG 52:1 in HFD females and the decrease of TG 54:7 in HFD males (Figure S10).

#### Fecal Acylcarnitine Profile

**Figure 10:**
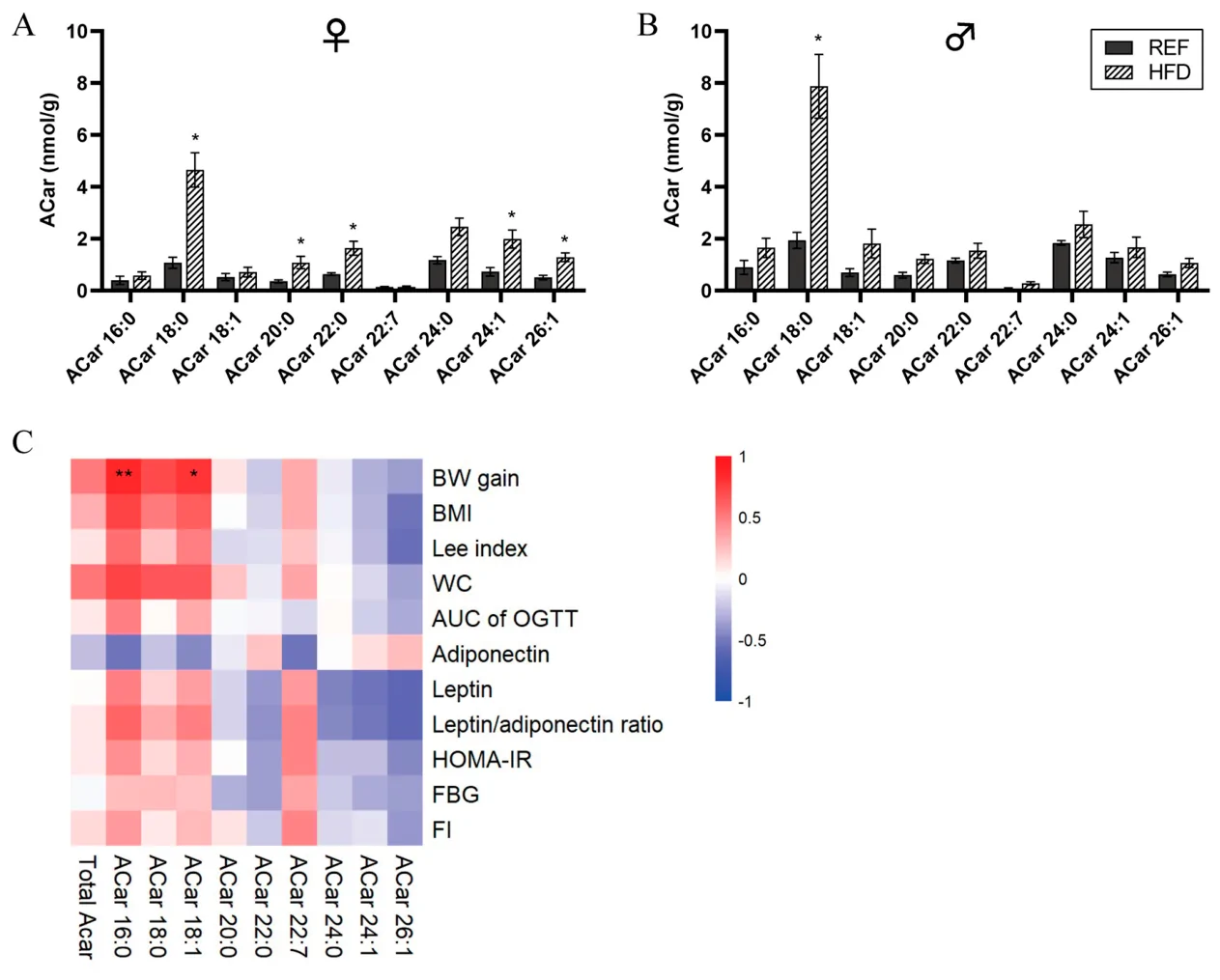
Fecal acylcarnitine (ACar) species. Fecal concentration of ACar species in (**A**) female rats and (**B**) male rats. Data are expressed as mean ± SEM. Statistical significance was assessed by *t*-test of transformed data with FDR correction (*p*-adj < 0.05; *n* = 7/group and sex). (**C**) Heatmap showing correlations between fecal ACar species with physiological and metabolic variables associated with obesity in the HFD group (*n* = 14). Spearman correlation coefficients are represented by color, with blue indicating negative correlations and red indicating positive correlations. Statistically significant correlations after FDR correction are marked with asterisks: * *p*-adj < 0.05, ** *p*-adj < 0.01. BW gain, body weight gain; BMI, body mass index; WC, waist circumference; AUC OGTT, area under the curve of the oral glucose tolerance test; HOMA-IR, homeostatic model assessment of insulin resistance; FBG, fasting blood glucose; FI, fasting insulin.

A total of nine ACar species were detected in fecal samples (Figure 10). In females, HFD exposure led to higher concentrations of the ACar species ACar 18:0, ACar 20:0, ACar 22:0, ACar 24:1, and ACar 26:1, indicating a marked diet-induced accumulation of long-chain (LC) ACar in feces [26] (Figure 10A). In contrast, in males, only ACar 18:0 was significantly increased in HFD compared to REF animals, reaching levels higher than those observed in females (Figure 10B).

To further explore the metabolic relevance of fecal ACar concentrations, within-diet correlation analyses were performed. No significant associations were detected in the REF group after FDR correction (Figure S6). In contrast, within the HFD group, ACar 16:0 and ACar 18:1 were positively associated with BW gain, indicating that higher fecal concentrations of these species were associated with greater weight gain among HFD animals (Figure 10C; Table S8).

## 4. Discussion

Dietary fat intake is a major driver of metabolic alterations, and HFDs are widely used to model obesity-associated metabolic dysfunction, including disruptions in lipid metabolism [1,2]. Although HFD-induced alterations in circulating lipids are well documented, less attention has been given to how dietary fat affects lipid distribution across multiple biological compartments and the extent to which these alterations may yield complementary metabolic insights. In this context, feces represent a relevant but understudied matrix, as they can provide insights into intestinal lipid metabolism, dietary intake, and host–microbiota interactions [27]. Accordingly, this study sought to comprehensively characterize the effects of HFD exposure on the plasma and fecal lipidomes in rats, and to assess whether these responses differ between sexes.

Morphometric and metabolic characterization confirmed the establishment of a diet-induced metabolic phenotype. HFD-fed animals exhibited increased BW gain and BMI together with impaired glucose tolerance. These alterations were more pronounced in males, which also displayed a higher leptin/adiponectin ratio, consistent with previous reports where greater male susceptibility to HFD-induced metabolic dysfunction was reported [9,10,28,29]. No differences were observed in the Lee index, WC, FI, HOMA-IR, leptin, and adiponectin, suggesting that certain metabolic alterations may require longer HFD exposure or a different composition of HFD or more severe metabolic impairment to become evident [30,31,32,33,34]. These findings establish a relevant basis for interpreting the lipidomic alterations detected across the two biological compartments assessed in the study.

The plasma and fecal lipidomes displayed markedly different responses to HFD exposure. Although both matrices shared several lipid classes, plasma lipids underwent extensive remodeling at the species level, as demonstrated by PCA and volcano plot analyses. In contrast, the fecal lipidome remained comparatively stable, with only a limited number of lipid species showing significant alterations. Sex also had a modest but significant influence on plasma lipid composition, while fecal lipid profiles were largely unaffected. This divergence highlights the distinct sensitivity of these lipidomic compartments, with plasma reflecting rapid systemic metabolic adaptations to dietary fat intake, while fecal lipids may primarily reflect downstream processes related to intestinal lipid handling, absorption efficiency, and host–microbiota-mediated metabolism [27].

Further differences emerged when total lipid levels and class distributions were examined. Despite the extensive remodeling observed at the species level, total plasma lipids remained unchanged in response to HFD, suggesting that dietary fat induces compositional changes rather than net lipid accumulation in the circulation. This observation contrasts with the commonly reported increase in circulating lipid levels associated with HFD [35,36,37,38] and may indicate the activation of compensatory mechanisms, such as enhanced lipid clearance, storage in peripheral tissues, or redistribution across metabolic pathways [39,40,41]. In contrast, total fecal lipid levels were increased in HFD females, largely driven by higher fecal ChoE levels, the predominant lipid class detected in this matrix. Increased fecal lipid excretion has also been reported following high-fat feeding [42,43]. However, the higher fecal ChoE levels could also reflect differences in intestinal cholesterol handling, including differences in the fraction of dietary cholesterol that remains unabsorbed [44]. Thus, although increased fecal lipid excretion may contribute to the higher fecal lipid levels observed in HFD females, the present data do not allow us to distinguish between altered intestinal absorption and excretion.

At the class and subclass levels, HFD-induced alterations in the plasma lipidome were selective rather than widespread. Total PE levels increased, whereas LPC and LPE levels decreased, suggesting targeted remodeling of glycerophospholipid metabolism. These findings are in line with previous studies demonstrating that HFD feeding and obesity are associated with perturbations in phospholipid metabolism [45,46]. However, changes at the lipid class level are often inconsistent across studies and appear to depend on the specific lipid species affected and the underlying metabolic context [47,48]. In general, reductions in circulating lysophospholipids have been frequently reported in obesity and HFD models [46,49], whereas elevated PE levels have been linked to metabolic dysfunction and impaired metabolic health [50,51,52,53]. In addition, males displayed lower LPE levels than females, contrasting with observations reported in humans [54,55], as well as higher DG and TG, consistent with previous findings [54]. These results further support the existence of intrinsic sex-related differences in lipid metabolism and handling. In contrast, class-level changes in feces were limited. Reduced PE levels were observed in HFD-fed males, while ACar, a lipid subclass uniquely detected in this compartment, showed a marked increase, indicating that the fecal lipidome responds more selectively to dietary fat exposure than the plasma lipidome.

Among plasma sphingolipids, distinct patterns were observed for Cer and SM. Cer are key bioactive lipids involved in cell signaling pathways regulating proliferation, autophagy, or apoptosis and have been widely associated with metabolic dysfunction [56]. Importantly, their biological effects depend strongly on acyl-chain length, with shorter-chain Cer generally considered more detrimental than LC species [15]. In the present study, only four LC Cer species were detected [15]. Therefore, the present dataset does not allow conclusions regarding shorter-chain Cer species, including C16:0- and C18:0-containing Cer that have been extensively implicated in cardiometabolic disease [57]. Two of the detected LC Cer species were reduced exclusively in HFD females, and although several LC Cer species have previously been reported to increase in obesity and metabolic disorders [56,58,59,60], these observed reductions may reflect a potential compensatory or protective response in females. Furthermore, the lack of associations between these Cer and physiological or metabolic variables suggest that their contribution to the metabolic phenotype may have been limited under the reported condition in this model. In contrast, SM, which also play important roles in membrane structure and cell signaling [61], exhibit a more pronounced response to HFD. This was characterized by increases in saturated and monounsaturated species containing C17–C20 acyl chains and accompanied by decreases in longer-chain species (C23–C24). Increased SM levels have previously been reported in obesity and metabolic disorders and are associated with increased metabolic risk [61,62,63,64]. Moreover, SM serve as a reservoir for Cer [61], which may partially explain the coordinated alterations observed in some corresponding Cer and SM species. However, the within-diet correlation analysis did not identify significant associations between SM species and the assessed variables in HFD animals. Interestingly, some associations were detected within the REF group, including positive associations of saturated species containing C14–C16 acyl chains with leptin and the leptin/adiponectin ratio, and a negative association of SM 18:1;2/23:1 with BMI. These findings should be interpreted cautiously, as they were observed in the REF group and, therefore, do not directly support an association between HFD-induced SM remodeling and metabolic dysfunction. They may instead reflect inter-individual variation in sphingolipid metabolism under the REF diet, although the underlying biological significance of these relationships remains uncertain.

Plasma ChoE exhibited one of the most consistent responses to HFD across sexes. ChoE constitutes the main storage and transport form of cholesterol in circulating lipoproteins and has been linked to cardiovascular risk, with some studies showing increases following HFD exposure [65]. In the present study, HFD induced a shift in the circulating ChoE profile, characterized by increased abundance of saturated and monounsaturated species (ChoE 18:0 and ChoE 18:1) and reductions in unsaturated species, including ChoE 18:3 and ChoE 20:5, consistent with previous reports [66]. In contrast, the reductions in ChoE 14:0 and ChoE 16:1 differ from findings in other studies that have associated these species with metabolic dysfunction [67]. Analysis of FA composition of ChoE further demonstrated a significant reduction of *n*-3 PUFA ChoE species and a concomitant increase in the *n*-6/*n*-3 ratio under HFD conditions. This pattern likely reflects the lower dietary intake of *n*-3 PUFA and has commonly been associated with a more pro-inflammatory metabolic environment [68]. Notably, females exhibited higher baseline levels of *n*-3 levels and lower *n*-6/*n*-3 ratios than males, suggesting a comparatively more favorable lipid profile prior to HFD feeding. Correlation analyses further revealed positive associations between several ChoE species and obesity-related variables, including BW gain, BMI, WC, leptin, and the leptin-to-adiponectin ratio. Notably, ChoE 14:0 and ChoE 16:1, which were reduced under HFD conditions, were positively associated with some of these variables, indicating that changes in their abundance did not necessarily parallel their relationships with the metabolic phenotype. Conversely, several species showed associations in both dietary groups, suggesting that the relationship between individual ChoE species and metabolic traits is species-dependent and not exclusively driven by HFD-induced changes in abundance.

Within plasma glycerophospholipids, the PC class underwent a pronounced remodeling in response to HFD, with a substantial number of species being altered in both sexes. PC are major structural components of cellular membranes, but they also play important roles in lipid metabolism and serve as precursors for numerous signaling molecules [69]. Notably, most of the observed changes involved reductions in both saturated and unsaturated PC species, with a high degree of overlap between females and males, suggesting a consistent diet-induced shift in PC composition. Interestingly, correlation analyses revealed that within the HFD group, several PC species positively associated with obesity-related variables were either unchanged or reduced under HFD conditions. This apparent discrepancy complicates the interpretation of their biological significance and suggests that alterations in PC abundance alone may not fully capture their contribution to metabolic dysfunction. Furthermore, interpretation of these findings is constrained by the analytical approach, which did not allow resolution of the precise FA composition of individual PC species. Consequently, key features, such as FA saturation and the *n*-6/*n*-3 FA ratio, could not be determined, despite their known influence on the biological functions of phospholipids [70]. Thus, reductions in specific PC species under HFD conditions should be interpreted with caution, as they do not necessarily indicate an improvement in metabolic health.

LPC, the predominant lysoglycerophospholipids in circulation, are primarily generated from the cleavage of PC and play important roles in lipid transport, energy metabolism, and cell signaling. Consistent with previous reports, HFD-induced obesity is commonly associated with reduced circulating LPC levels, although this may vary according to FA composition of individual species [71]. In our study, HFD resulted in a consistent reduction across a broad range of LPC species in both sexes, in accordance with the previous report. Furthermore, HFD feeding increased the *n*-6/*n*-3 LPC ratio, indicating not only a global reduction in LPC abundance but also a shift toward a more pro-inflammatory lipid environment, similar to that observed in ChoE. This change likely reflects alterations in dietary FA intake and lipid remodeling pathways associated with metabolic dysfunction. Despite these marked changes in LPC species, they did not correlate with metabolic parameters in this study, contrary to previous reports [72]. The lack of association observed here may reflect differences in experimental design, dietary composition, animal characteristics, or the complex and context-dependent roles of LPC species in metabolic regulation. Collectively, these results suggest that while LPC remodeling is a robust feature of the HFD response, its relationship with metabolic health may not be straightforward and could depend on broader metabolic and lipidomic network interactions.

Plasma PE showed a limited but biologically relevant response to HFD, characterized by increased total PE levels and PE 36:2 in both sexes, consistent with previous evidence linking PE accumulation to metabolic dysfunction [50,51,52,53]. As major membrane phospholipids involved in structural integrity, cellular metabolism, and signaling transduction [73], alterations in PE may have important physiological consequences. In the present study, PE 36:4 was positively associated with the leptin/adiponectin ratio within the REF group, although this species was not altered by HFD, suggesting that their metabolic relevance may extend beyond simple changes in abundance. Ether-linked PE species displayed a distinct pattern, with PE-O 40:7 increased specifically in HFD males. Although the physiological functions of ether-linked lipids remain incompletely understood and their responses to metabolic disturbances appear variable across studies, these findings may reflect a compensatory adaptation to HFD-induced oxidative stress, in agreement with previous reports showing inconsistent changes in this lipid subclass under obesogenic conditions [74]. In contrast to PE, LPE exhibited lower total LPE abundance and reduced LPE 16:0 under HFD conditions. Moreover, positive correlations with adiponectin were found for LPE 18:2 within HFD animals. However, levels of LPE 18:2, whose reduced serum levels have been linked to higher LDL-cholesterol and hypercholesterolemia in humans [75], did not change due to HFD. These findings support a potential beneficial role of LPE in metabolic homeostasis, in agreement with emerging evidence indicating that they may exert anti-inflammatory and metabolically beneficial effects, although the underlying mechanisms remain to be fully elucidated [76].

Regarding plasma glycerolipids, DG showed a modest response to HFD, with only a limited number of species being increased. As key intermediates in lipid metabolism and signaling pathways, DG have frequently been linked to metabolic dysfunction, insulin resistance, and related disorders, with elevated levels frequently reported in response to HFD feeding [16,49]. In the present study, DG 34:1 was positively associated with BW gain within the REF group, whereas no significant associations were detected within HFD animals. Thus, the present correlation analysis provides limited evidence for a direct relationship between HFD-induced DG remodeling and the metabolic variables assessed, despite the known involvement of DG in metabolic dysfunction. In contrast, TG underwent extensive remodeling at the species level, particularly in females, although the overall direction of change was broadly similar between sexes. The simultaneous increase of some TG and decrease of others indicates a substantial reorganization of TG composition rather than simple global accumulation of circulating TG. Such remodeling likely reflects alterations in FA availability, esterification pathways, and lipid storage dynamics induced by HFD. Despite the well-established association between elevated TG, obesity, and metabolic disorders [16], neither total TG nor individual TG species showed significant correlations with the metabolic parameters evaluated in this study. This suggests that these changes may primarily reflect adaptations in lipid storage and redistribution rather than direct relationships with the metabolic traits measured. A similar consideration to PC applies to DG and TG alterations, as the FA composition of individual species could not be resolved. Given the strong influence of FA composition on the biological effects of DG and TG [16], more detailed structural lipidomics analyses may help identify the glycerolipid species closely associated with metabolic dysfunction.

Among fecal lipids, ACar was the only subclass consistently altered by HFD. ACar are intermediates of FA oxidation that facilitate the transport of FA into mitochondria for β-oxidation. Their accumulation is often considered a marker of incomplete FA oxidation or metabolic overflow [77]. Nine ACar species, all considered LC [26], were detected. Among these, five saturated (ACar 18:0, 20:0, and 22:0) and monounsaturated species (ACar 24:1 and 26:1) were significantly increased in HFD females. In males, only ACar 18:0 reached statistical significance, although the remaining species followed a similar upward trend, indicating a broadly comparable response across sexes. Accordingly, total fecal ACar levels were elevated in both sexes, with higher levels observed in males. This sex difference has previously been reported for circulating carnitine levels both in humans and rodents [78] and, in the present study, was largely driven by the marked increase in ACar 18:0 in males.

The enrichment of LC ACar in feces is particularly relevant, as these species have been widely associated with impaired FA oxidation and metabolic dysfunction in obesity and type 2 diabetes, where they are considered markers of incomplete β-oxidation [79]. Similar increases in fecal ACar have been reported in patients with inflammatory bowel disease and cirrhosis, and have been associated to gut dysbiosis, bile acid alterations, and impaired intestinal function [77,80]. Several non-mutually exclusive processes may contribute to this accumulation, including impaired intestinal FA absorption, increased luminal secretion, or microbial modulation of ACar, which is increasingly recognized as an important determinant of host metabolic homeostasis [77]. The increase in very LC species (>C22), which are oxidized in peroxisomes [81], may further suggest alterations in intestinal lipid handling involving both mitochondrial and peroxisomal FA oxidation.

Importantly, although ACar were detected in fecal samples, they were not detected in plasma using the same lipidomics workflow. This difference should not be interpreted as evidence that ACar are absent from plasma or that they are selectively altered in feces. Rather, matrix-dependent analytical characteristics may have contributed to their differential detectability. In addition, the lipid species included in the semi-targeted analysis were generated independently for each matrix based on compounds detected and successfully annotated. Consequently, the present data do not allow a direct comparison of circulating and fecal ACar concentrations or conclusions regarding their relative biological abundance between matrices. Nevertheless, fecal ACar may be influenced by processes occurring within or directed toward the intestinal lumen. Hepatically produced ACar can be secreted into bile and subsequently delivered to the intestine, while gut microorganisms can utilize carnitine and different acylcarnitine species [7,77,82]. Thus, the fecal accumulation observed in the present study may reflect altered intestinal secretion and/or microbial handling of ACar, rather than necessarily representing systemic mitochondrial β-oxidation overload.

Within-diet correlation analyses further showed that fecal ACar 16:0 and ACar 18:1 were positively associated with BW gain in HFD animals, whereas no significant associations were detected in REF animals. These findings indicate that higher fecal concentrations of specific ACar were associated with greater weight gain among HFD animals, supporting their potential relevance to the metabolic response to an obesogenic diet. Previous studies have also linked fecal ACar 16:0, 18:0, and 18:1 with obesity progression [79], while circulating ACar 18:1 has been linked to prediabetes and type 2 diabetes [83]. However, given the limited number of significant associations identified in the present study, these findings should be interpreted as evidence of a relationship between specific fecal ACar and BW gain under HFD conditions, rather than as evidence of a generalized association between fecal ACar and metabolic dysfunction.

Overall, the consistent accumulation of LC ACar in feces, together with their association with BW gain in HFD animals, supports their potential value as indicators of altered host–gut lipid metabolism. These findings highlight the added value of fecal lipidomics in capturing metabolic features that may not be reflected in the plasma lipid profile. To the best of our knowledge, studies investigating fecal ACar in HFD-induced obesity in rats remain limited, and the present results provide novel evidence for their accumulation and modulation under the studied conditions.

Several limitations should be considered when interpreting the present findings. The animals were 8 weeks old at the beginning of the intervention and approximately 20 weeks old at sacrifice, corresponding to the young adult/adult developmental range in rats [84]; however, sexual maturity was not specifically assessed, and extrapolation of these findings to adult obesity should, therefore, be made cautiously. In females, the estrous cycle was not controlled for or incorporated as an experimental factor—fluctuations in ovarian hormones, particularly estrogens, can influence lipid and glucose metabolism [85] and may, therefore, have contributed to biological variability in sex-specific metabolic and lipidomic responses. Thus, some of the observed sex-related variability may reflect differences in hormonal status, which should be considered when interpreting and reproducing these findings. The relatively modest sample size (n = 7/group) and the large number of lipid species comparisons should also be considered, as the previous power calculation did not account for multiple testing or the potential loss of power associated with FDR correction. Thus, the absence of widespread diet × sex interactions should be interpreted cautiously, as small-to-moderate interaction effects may have remained undetected. Furthermore, the semi-targeted, semiquantitative LC-MS approach and the use of positive electrospray ionization only may have limited lipid coverage, particularly for lipid classes that are more favorably detected in negative ionization mode, such as PS. Potential variability related to sample processing, the use of rats, and a single sampling time point may have further limited the detection of species-specific, temporal, and additional lipidomic changes and variability. Finally, future studies integrating lipidomics with microbiome profiling will be required to clarify the origin and biological significance of the fecal LC ACar identified in this study.

## 5. Conclusions

Overall, this study demonstrates that HFD induces extensive remodeling of the plasma lipidome across multiple lipid classes, largely driven by alterations at the individual species level, while the fecal lipidome displays a more selective but biologically meaningful response. Among fecal lipids, LC ACar in feces emerged as a distinctive feature of the fecal lipidome, showing consistent accumulation under HFD conditions. Furthermore, fecal ACar 16:0 and ACar 18:1 were positively associated with BW gain within the HFD group, supporting their potential relevance as indicators of altered host–gut lipid metabolism. These findings further highlight the value of fecal lipidomics as a complementary approach to plasma profiling, providing insights into intestinal and host–gut metabolic processes that may not be captured by systemic analysis alone. Sex emerged as a significant determinant of the plasma lipidome but exerted only a limited influence on the fecal lipid profile, indicating compartment-specific regulation of lipid metabolism. However, the effects of diet and sex appeared largely independent, as HFD elicited broadly similar lipidomic responses in both sexes, despite males exhibiting a more pronounced metabolic phenotype. Together, these results demonstrate the complex and lipid-class-specific nature of metabolic adaptations to HFD and underscore the value of integrating multiple biological matrices and considering sex as an intrinsic biological variable in lipidomic and metabolic research. Further studies are warranted to investigate the mechanisms underlying fecal ACar accumulation and their potential links with host metabolism, intestinal physiology, and gut microbiota in obesity and related metabolic conditions.

## Figures and Tables

**Table 1:** Morphometric and metabolic variables of the study groups.

	REF		HFD		*p*-Value		
	Females	Males	Females	Males	D	S	D × S
BW gain (% )	34.48 ± 3.91	61.78 ± 8.21	46.33 ± 2.50	86.12 ± 6.16	0.004	<0.001	ns
BMI (g/cm^2^)	0.61 ± 0.01	0.80 ± 0.02	0.63 ± 0.02	0.91 ± 0.04	0.021	<0.001	ns
Lee index ((g^1/3^/cm) × 10^3^)	304.63 ± 2.90	315.84 ± 3.68	308.63 ± 4.85	328.72 ± 6.44	ns	0.001	ns
WC (cm)	16.94 ± 0.55	22.11 ± 0.48	17.20 ± 0.44	23.49 ± 0.98	ns	<0.001	ns
AUC of the OGTT	16,737.00 ± 680.51	15,996.57 ± 403.77	17,165.71 ± 774.78	18,365.71 ± 640.60	0.039	ns	ns
FBG (mg/dL)	118.29 ± 4.24	107.43 ± 4.52	108.43 ± 5.25 *	118.43 ± 4.62	ns	ns	0.013
FI (µIU/mL)	13.05 ± 4.53	23.37 ± 9.34	8.74 ± 1.00	16.76 ± 5.76	ns	ns	ns
HOMA-IR	3.86 ± 1.38	6.44 ± 2.76	2.33 ± 0.26	5.16 ± 2.01	ns	ns	ns
Leptin (pg/mL)	444.02 ± 85.74	1330.28 ± 260.06	482.84 ± 131.63	1664.21 ± 331.87	ns	<0.001	ns
Adiponectin (ng/mL)	17,369.61 ± 2047.62	12,753.66 ± 1881.84	17,281.20 ± 3296.36	7654.32 ± 940.95	ns	<0.001	ns
Leptin/adiponectin ratio	0.03 ± 0.01	0.11 ± 0.02	0.03 ± 0.00	0.23 ± 0.05 *	0.001	<0.001	<0.001

Data are expressed as mean ± SEM. Statistical differences: *p*-values for the main effects of diet, sex, and their interaction were obtained by two-way ANOVA or ART-ANOVA, as appropriate, and are shown in the table (*n* = 7 per group and sex). When a significant diet × sex interaction was detected (*p* < 0.05), simple effects were analyzed. * *p* < 0.05 vs. the corresponding REF group. BW gain: body weight gain; BMI: body mass index; WC: waist circumference; AUC of the OGTT: area under the curve of the oral glucose tolerance test; FBG: fasting blood glucose; FI: fasting insulin; HOMA-IR: homeostatic model assessment of insulin resistance; D, diet; S, sex; D × S, diet × sex interaction; ns, no statistical significance detected.

## Data Availability

The datasets generated and/or analyzed during the current study are available from the corresponding author upon reasonable request.

## Supplementary Materials

The following supporting information can be downloaded at: https://www.mdpi.com/article/10.3390/nu18183014/s1. Figure S1: Plasma sphingolipid correlation study in the HFD group. Figure S2: Plasma phosphatidylcholine (PC) correlation study in the HFD group. Figure S3: Plasma lysophosphatidylcholine (LPC) correlation study within dietary groups. Figure S4: Plasma glycerolipid correlation study in the HFD group. Figure S5: Plasma triacylglycerol (TG) correlation study within dietary groups. Figure S6: Fecal acylcarnitine (ACar) correlation study in the REF group. Figure S7: Fecal sphingolipids. Figure S8: Fecal cholesteryl esters (ChoE). Figure S9: Fecal glycerophospholipids. Figure S10: Fecal glycerophospholipids. Table S1: Composition of experimental diets. Table S2: Analytical variability of the lipidomic measurements based on pooled quality-control samples. Table S3: Internal standards used for normalization of plasma lipid classes/subclasses. Table S4: Internal standards used for normalization of fecal lipid classes/subclasses. Table S5: Number of lipid species identified and significantly affected by diet, sex, and diet × sex interaction in plasma and feces. Table S6: Significantly altered lipid species by HFD intake influenced by diet, sex, or their interaction. Table S7: Differential plasma lipid species across comparisons. Table S8: Significant FDR-adjusted Spearman correlations between lipid species and physiological variables within dietary groups.

## Abbreviations

The following abbreviations are used in this manuscript:

Acar	acylcarnitine
ART-ANOVA	aligned rank transform for non-parametric factorial ANOVA
AUC	area under the curve
BMI	body mass index
BW	body weight
ChoE	cholesteryl ester
Cer	ceramide
DG	diacylglycerol
FA	fatty acid
FBG	fasting blood glucose
FC	fold change
FDR	false discovery rate
FI	fasting insulin
HFD	high-fat diet
HOMA-IR	homeostasis model assessment insulin resistance
LC-MS	liquid chromatography coupled with mass spectrometry
LC	long-chain
LPC	lysophosphatidylcholine
LPE	lysophosphatidylethanolamine
MG	monoacylglycerol
OGTT	oral glucose tolerance test
PC	phosphatidylcholine
PC-O	ether-linked phosphatidylcholine
PCA	principal component analysis
PE	phosphatidylethanolamine
PE-O	ether-linked phosphatidylethanolamine
PERMANOVA	permutational multivariate analysis of variance
PS	phosphatidylserine
PUFA	polyunsaturated fatty acid
REF	reference diet
SEM	standard error of the mean
SM	sphingomyelin
TG	triacylglycerol
UHPLC-qTOF	ultra-high-performance liquid chromatography system coupled to quadrupole time-of-flight mass spectrometry
WC	waist circumference
