# Supplementary material for: Sex-Dependent Lipidomic Remodeling in Plasma and Feces Following High-Fat Diet Intake in Adult Rats
Source: Nutrients. 2026 Sep 15;18(18):3014. doi: 10.3390/nu18183014 (PMC13610511; doi:10.3390/nu18183014)
Supplement: Supplementary file 1 [file nutrients-18-03014-s001.zip › Supplementary material_nutrients_lipidomics.pdf]

Supplementary material

Table S1. Composition of experimental diets.

	REF diet	HFD diet
**Components**	**g/kg of diet**	
Casein	140	245
L-cystine	1.8	3.5
Corn Starch	465.692	85
Maltodextrin	155	115
**Sucrose**	**100**	**200**
**Lard**	**-**	**195**
Soybean Oil	40	30
Cellulose	50	58
Mineral Mix, AIN-93M-MX (94049)	35	-
Mineral Mix, AIN-93G-MX (94046)	-	43
Calcium Phosphate, dibasic	-	3.4
Vitamin Mix, AIN-93-VX (94047)	10	19
Choline Bitartrate	2.5	3
TBHQ, antioxidant	0.008	-
Red Food Color	-	0.1
**Nutrient information**	**%kcal of diet from**	
Protein	13.7	19.0
Carbohydrate	75.9	36.2
Fat	10.3	44.8
**Kcal/g**	**3.6**	**4.6**

Table S2. Analytical variability of the lipidomic measurements based on pooled quality-control samples.

	Lipid class	Nº of lipid species	Median RSD (%)	Range of RSD (%)
**Plasma**	Cer	4	8.95	5.1–10.5
	ChoE	11	11.10	3.7–19.3
	DG	4	3.85	2.5–9.8
	LPC	24	4.05	1.5–17.6
	LPC-O	4	11.15	3.8–19.4
	LPE	3	12.40	12.1–18.1
	MG	1	6.20	6.2–6.2
	PC	40	5.55	1.3–18.8
	PC-O	14	12.80	3.0–23.3
	PE	4	9.20	6.0–22.2
	PE-O	3	12.50	9.3–25.1
	SM	23	9.80	1.4–25.2
	TG	35	9.20	2.9–23.1
**Feces**	ACar	9	10.10	6.5–19.4
	ChoE	9	10.00	4.5–16.1
	Cer	13	13.60	7.1–29.2

DG	7	15.50	8.2–21.4
LPC	13	11.90	6.3–26.1
LPE	1	8.00	8.0–8.0
PC	28	10.80	2.9–22.9
PE	11	19.00	7.4–25.3
PS	4	16.65	8.2–25.8
SM	9	16.50	8.3–25.2
TG	22	18.70	7.7–23.2

The relative standard deviation (RSD) (%) was calculated across eight pooled QC injections performed throughout the analytical sequence for each matrix. RSD values represent analytical variability for each detected lipid species. Median RSD and range are reported for each lipid class/subclass.

Table S3. Internal standards used for normalization of plasma lipid classes/subclasses.

Lipid class/subclass	Nº of lipid species	Internal standard used
**Cer**	4	d18:1-18:1(d9) SM*
**ChoE**	11	18:1(d7) ChoE
**DG**	4	15:0-18:1(d7) DG
**LPC**	24	18:1(d7) Lyso PC
**LPC-O**	4	18:1(d7) Lyso PC*
**LPE**	3	18:1(d7) Lyso PE
**MG**	1	18:1(d7) MG
**PC**	40	15:0-18:1(d7) PC
**PC-O**	14	15:0-18:1(d7) PC*
**PE**	4	15:0-18:1(d7) PE
**PE-O**	3	15:0-18:1(d7) PE*
**SM**	23	d18:1-18:1(d9) SM
**TG**	35	15:0-18:1(d7)-15:0 TG

Table S4. Internal standards used for normalization of fecal lipid classes/subclasses.

Lipid class/subclass	Nº of lipid species	Internal standard used
**ACar**	9	18:1(d7) Lyso PC*
**ChoE**	9	18:1(d7) ChoE
**Cer**	13	d18:1-18:1(d9) SM*
**DG**	7	15:0-18:1(d7) DG
**LPC**	13	18:1(d7) Lyso PC
**LPE**	1	18:1(d7) Lyso PE
**PC**	28	15:0-18:1(d7) PC
**PE**	11	15:0-18:1(d7) PE
**PS**	4	15:0-18:1(d7) PS
**SM**	9	d18:1-18:1(d9) SM
**TG**	22	15:0-18:1(d7)-15:0 TG

Table S5. Number of lipid species identified and significantly affected by diet, sex, and diet × sex interaction in plasma and feces.

	Lipid class/subclass	Detected/tested species (nº)	Diet effect (nº species)	Sex effect (nº species)	Interaction effect (nº species)
**Plasma**	Ceramides (Cer)	4	0	0	0
	Cholesterol esters (ChoE)	11	6	6	0
	Diacylglycerols (DG)	4	3	3	0
	Lysophosphatidylcholines (LPC)	28	18	2	0
	Lysophosphatidylethanolamines (LPE)	3	1	2	0
	Monoacylglycerols (MG)	1	0	0	0
	Phosphatidylcholines (PC)	54	34	28	0
	Phosphatidylethanolamines (PE)	7	2	1	0
	Sphingomyelins (SM)	23	8	6	0
	Triacylglycerols (TG)	35	27	10	0
	**TOTAL**	**170**	**99**	**58**	**0**
**Feces**	Acylcarnitines (Acar)	9	2	0	0
	Ceramides (Cer)	13	0	0	0
	Cholesterol esters (ChoE)	9	0	0	0
	Diacylglycerols (DG)	7	0	0	0
	Lysophosphatidylcholines (LPC)	13	0	0	0
	Lysophosphatidylethanolamines (LPE)	1	0	0	0
	Phosphatidylcholines (PC)	28	0	0	0
	Phosphatidylethanolamines (PE)	11	0	0	0
	Phosphatidylserines (PS)	4	0	0	0
	Sphingomyelins (SM)	9	0	0	0
	Triacylglycerols (TG)	22	4	0	0
	**TOTAL**	**126**	**6**	**0**	**0**

Values represent the number of lipid species identified in each lipid class and the number of species showing a significant effect of diet, sex, or diet × sex interaction. Significancy was determined based on Benjamini–Hochberg FDR-adjusted *p*-values < 0.05.

Table S6. Significantly altered lipid species by HFD intake influenced by diet, sex or their interaction.

	Species	Effect	REF	HFD	FC (HFD/REF)	Diet (*p*-adj)	Females	Males	FC (F/M)	Sex (*p*-adj)	D × S (*p*-adj)
Plasma	**ChoE 14:0**	D; S	1.53 ± 0.20	0.71 ± 0.13	0.47	0.000	0.78 ± 0.11	1.46 ± 0.23	0.54	0.000	0.636
	**ChoE 16:0**	S	44.87 ± 2.47	47.77 ± 4.01	1.06	0.566	38.77 ± 2.03	53.87 ± 3.10	0.72	0.011	0.689
	**ChoE 16:1**	D; S	61.19 ± 8.22	19.88 ± 2.79	0.34	0.000	25.71 ± 3.91	55.36 ± 9.58	0.41	0.000	0.874
	**ChoE 18:0**	D	3.03 ± 0.25	6.49 ± 0.38	1.55	0.000	4.66 ± 0.53	4.87 ± 0.61	0.87	0.644	0.887
	**ChoE 18:1**	D; S	132.57 ± 9.93	233.06 ± 29.67	1.76	0.000	122.21 ± 6.70	243.42 ± 27.52	0.50	0.000	0.113
	**ChoE 18:3**	D	148.69 ± 11.30	78.59 ± 5.74	0.53	0.000	112.36 ± 12.94	114.92 ± 13.49	0.98	0.783	0.636
	**ChoE 20:5**	D; S	400.06 ± 32.64	112.06 ± 9.65	0.28	0.000	299.06 ± 50.03	213.06 ± 39.51	1.40	0.003	0.973
	**ChoE 22:6**	S	611.68 ± 54.75	548.89 ± 45.92	0.90	0.477	670.56 ± 49.08	490.00 ± 39.94	1.37	0.004	0.784
	**DG 34:1**	D	1.10 ± 0.10	1.52 ± 0.14	1.38	0.000	1.21 ± 0.12	1.40 ± 0.14	0.86	0.284	0.479
	**DG 34:2**	D; S	1.61 ± 0.21	1.13 ± 0.14	0.70	0.008	1.08 ± 0.11	1.66 ± 0.22	0.65	0.019	0.636
	**DG 36:2**	D; S	1.49 ± 0.17	2.19 ± 0.26	1.47	0.000	1.53 ± 0.15	2.15 ± 0.28	0.71	0.019	0.874
	**DG 36:3**	S	1.77 ± 0.24	1.73 ± 0.21	0.98	0.811	1.32 ± 0.12	2.18 ± 0.25	0.61	0.002	0.891
	**LPC 14:0**	D	1.31 ± 0.09	0.64 ± 0.05	0.49	0.000	0.86 ± 0.08	1.09 ± 0.14	0.80	0.174	0.947
	**LPC 15:0**	D	0.74 ± 0.04	0.46 ± 0.02	0.62	0.000	0.55 ± 0.04	0.64 ± 0.05	0.86	0.324	0.985
	**LPC 16:0**	D	100.39 ± 5.19	67.82 ± 3.92	0.68	0.000	77.94 ± 5.87	90.26 ± 6.54	0.86	0.235	0.636
	**LPC 16:0 e**	D	0.56 ± 0.02	0.43 ± 0.02	0.76	0.001	0.48 ± 0.02	0.50 ± 0.03	0.96	0.822	0.973
	**LPC 16:1**	D	6.00 ± 0.77	1.66 ± 0.17	0.28	0.000	3.03 ± 0.48	4.63 ± 1.01	0.65	0.136	0.650
	**LPC 16:1 e**	D	0.17 ± 0.00	0.14 ± 0.01	0.82	0.022	0.16 ± 0.01	0.16 ± 0.01	0.96	0.760	0.973
	**LPC 18:0**	S	92.91 ± 7.72	105.74 ± 5.62	1.14	0.065	116.33 ± 4.82	82.32 ± 5.46	1.41	0.000	0.479
	**LPC 18:0 e**	D	0.17 ± 0.01	0.21 ± 0.01	1.29	0.004	0.20 ± 0.01	0.18 ± 0.01	1.14	0.051	0.737
	**LPC 18:2**	D	51.59 ± 4.51	35.02 ± 3.33	0.68	0.002	44.35 ± 3.45	42.26 ± 5.47	1.05	0.177	0.636
	**LPC 18:3**	D	12.05 ± 1.01	6.52 ± 0.50	0.54	0.000	8.12 ± 0.89	10.45 ± 1.20	0.78	0.077	0.701
	**LPC 19:0**	D	0.60 ± 0.02	0.42 ± 0.02	0.69	0.000	0.50 ± 0.04	0.52 ± 0.03	0.97	0.775	0.701
	**LPC 20:0**	D; S	0.40 ± 0.02	0.28 ± 0.01	0.69	0.000	0.36 ± 0.03	0.32 ± 0.02	1.14	0.031	0.433
	**LPC 20:3**	D	2.34 ± 0.30	1.31 ± 0.11	0.56	0.000	1.63 ± 0.18	2.03 ± 0.32	0.80	0.533	0.858
	**LPC 20:5**	D	5.13 ± 0.59	3.18 ± 0.30	0.62	0.002	4.04 ± 0.36	4.28 ± 0.67	0.94	0.550	0.701
	**LPC 22:5**	D	0.54 ± 0.05	0.32 ± 0.02	0.59	0.000	0.41 ± 0.04	0.45 ± 0.06	0.91	0.961	0.973
	**LPC 22:6**	D	5.76 ± 0.48	4.55 ± 0.39	0.79	0.041	5.49 ± 0.44	4.83 ± 0.48	1.14	0.077	0.962
	**LPC 24:0**	D	0.29 ± 0.01	0.19 ± 0.01	0.66	0.000	0.25 ± 0.02	0.24 ± 0.01	1.04	0.295	0.636

	**LPC-O 16:1p**	D	0.17 ± 0.00	0.14 ± 0.01	0.81	0.019	0.15 ± 0.01	0.16 ± 0.01	0.96	0.724	0.968
	**LPC-O 24:1p**	D	0.03 ± 0.00	0.02 ± 0.00	0.70	0.028	0.02 ± 0.00	0.03 ± 0.00	0.89	0.597	0.962
	**LPE 16:0**	D; S	1.73 ± 0.09	1.13 ± 0.09	0.65	0.000	1.61 ± 0.11	1.25 ± 0.11	1.29	0.000	0.636
	**LPE 18:2**	S	1.12 ± 0.10	0.99 ± 0.12	0.88	0.255	1.26 ± 0.09	0.86 ± 0.10	1.47	0.001	0.740
	**PC 30:0**	D	0.98 ± 0.07	0.52 ± 0.02	0.54	0.000	0.70 ± 0.06	0.80 ± 0.10	0.88	0.746	0.858
	**PC 30:1**	D; S	0.35 ± 0.05	0.13 ± 0.01	0.37	0.000	0.20 ± 0.03	0.29 ± 0.05	0.69	0.017	0.973
	**PC 31:0**	D	0.48 ± 0.03	0.26 ± 0.01	0.54	0.000	0.36 ± 0.03	0.38 ± 0.04	0.94	0.903	0.973
	**PC 32:0**	D	9.93 ± 0.76	7.12 ± 0.37	0.72	0.008	7.94 ± 0.66	9.11 ± 0.73	0.87	0.501	0.947
	**PC 32:1**	D; S	9.41 ± 1.47	2.63 ± 0.25	0.28	0.000	4.32 ± 0.68	7.72 ± 1.76	0.56	0.003	0.701
	**PC 32:2**	D; S	1.86 ± 0.26	0.71 ± 0.06	0.38	0.000	0.94 ± 0.12	1.62 ± 0.30	0.58	0.000	0.701
	**PC 33:0**	D	0.48 ± 0.02	0.38 ± 0.02	0.80	0.004	0.41 ± 0.02	0.45 ± 0.03	0.92	0.886	0.779
	**PC 33:1**	D; S	0.90 ± 0.08	0.75 ± 0.05	0.83	0.015	0.69 ± 0.03	0.96 ± 0.08	0.71	0.001	0.980
	**PC 33:2**	D	1.03 ± 0.10	0.61 ± 0.04	0.59	0.000	0.72 ± 0.06	0.93 ± 0.12	0.77	0.064	0.973
	**PC 34:0**	D	4.74 ± 0.37	5.53 ± 0.23	1.17	0.042	5.45 ± 0.34	4.82 ± 0.29	1.13	0.077	0.871
	**PC 34:1**	S	57.09 ± 4.95	51.84 ± 4.01	0.91	0.146	44.97 ± 2.50	63.95 ± 4.64	0.70	0.000	0.208
	**PC 34:2**	D; S	166.49 ± 11.35	122.43 ± 8.23	0.74	0.000	130.22 ± 11.11	158.70 ± 10.79	0.82	0.048	0.636
	**PC 34:3**	D; S	5.63 ± 0.70	2.50 ± 0.17	0.44	0.000	3.32 ± 0.39	4.80 ± 0.81	0.69	0.014	0.847
	**PC 34:4**	D; S	1.42 ± 0.23	0.71 ± 0.12	0.50	0.000	0.63 ± 0.08	1.50 ± 0.23	0.42	0.000	0.894
	**PC 34:5**	D; S	0.20 ± 0.03	0.08 ± 0.01	0.41	0.000	0.10 ± 0.01	0.18 ± 0.03	0.55	0.000	0.962
	**PC 35:1**	D	0.85 ± 0.04	1.04 ± 0.05	1.22	0.002	0.89 ± 0.04	1.00 ± 0.05	0.89	0.475	0.891
	**PC 35:3**	D; S	0.79 ± 0.08	0.59 ± 0.04	0.75	0.007	0.57 ± 0.05	0.80 ± 0.08	0.71	0.004	0.954
	**PC 35:4**	D; S	0.27 ± 0.03	0.21 ± 0.02	0.79	0.004	0.19 ± 0.01	0.29 ± 0.03	0.65	0.000	0.701
	**PC 36:0**	S	1.61 ± 0.18	1.92 ± 0.11	1.19	0.060	2.08 ± 0.12	1.45 ± 0.14	1.43	0.004	0.619
	**PC 36:1**	D; S	19.10 ± 1.61	23.35 ± 1.16	1.22	0.008	22.97 ± 1.04	19.48 ± 1.76	1.18	0.011	0.170
	**PC 36:2**	S	157.44 ± 9.41	169.73 ± 9.33	1.08	0.166	179.05 ± 9.03	148.12 ± 7.93	1.21	0.002	0.636
	**PC 36:3**	D; S	49.67 ± 5.02	36.70 ± 2.94	0.74	0.000	33.78 ± 2.59	52.59 ± 4.47	0.64	0.000	0.886
	**PC 36:4**	D; S	167.81 ± 13.44	140.93 ± 15.12	0.84	0.004	115.43 ± 10.01	193.31 ± 10.17	0.60	0.000	0.085
	**PC 36:5**	D; S	19.64 ± 1.79	12.69 ± 1.03	0.65	0.000	14.06 ± 1.56	18.26 ± 1.74	0.77	0.019	0.636
	**PC 37:2**	D	1.24 ± 0.08	0.80 ± 0.04	0.65	0.000	0.97 ± 0.09	1.07 ± 0.09	0.91	0.606	0.581
	**PC 37:4**	S	3.13 ± 0.20	3.38 ± 0.33	1.08	0.508	2.66 ± 0.19	3.85 ± 0.24	0.69	0.002	0.128
	**PC 38:1**	D; S	0.42 ± 0.03	0.58 ± 0.02	1.36	0.001	0.55 ± 0.03	0.45 ± 0.03	1.24	0.004	0.701
	**PC 38:2**	D	1.96 ± 0.15	2.23 ± 0.10	1.14	0.043	2.15 ± 0.14	2.04 ± 0.13	1.05	0.223	0.593

	**PC 38:4**	D	266.67 ± 17.94	328.43 ± 11.33	1.23	0.014	313.86 ± 12.30	281.24 ± 20.11	1.12	0.069	0.479
	**PC 38:5**	D; S	21.11 ± 3.57	13.95 ± 1.94	0.66	0.002	10.23 ± 0.96	24.82 ± 3.06	0.41	0.000	0.973
	**PC 38:6**	S	53.87 ± 5.17	45.87 ± 4.81	0.85	0.118	39.58 ± 4.17	60.16 ± 4.32	0.66	0.001	0.445
	**PC 38:7**	D; S	18.12 ± 1.58	14.75 ± 1.78	0.81	0.002	11.69 ± 1.12	21.18 ± 1.18	0.55	0.000	0.085
	**PC 40:4**	D; S	1.67 ± 0.15	1.97 ± 0.08	1.17	0.017	2.07 ± 0.06	1.57 ± 0.14	1.32	0.000	0.136
	**PC 40:6**	S	64.85 ± 8.48	67.05 ± 5.75	1.03	0.318	85.59 ± 5.40	46.31 ± 4.08	1.85	0.000	0.479
	**PC 40:8**	S	3.46 ± 0.48	3.41 ± 0.31	0.98	0.656	2.76 ± 0.16	4.12 ± 0.48	0.67	0.004	0.973
	**PC 40:9**	S	5.58 ± 0.44	4.90 ± 0.46	0.88	0.164	4.37 ± 0.42	6.12 ± 0.35	0.71	0.004	0.474
	**PC-O 34:3**	D	0.19 ± 0.01	0.14 ± 0.01	0.75	0.001	0.16 ± 0.01	0.18 ± 0.01	0.89	0.496	0.962
	**PC-O 36:1**	D	0.16 ± 0.01	0.21 ± 0.01	1.32	0.004	0.19 ± 0.01	0.17 ± 0.01	1.09	0.177	0.973
	**PC-O 36:4**	D; S	3.00 ± 0.22	2.47 ± 0.17	0.82	0.015	2.41 ± 0.17	3.06 ± 0.20	0.79	0.019	0.636
	**PC-O 38:4**	D	0.74 ± 0.07	1.14 ± 0.06	1.54	0.001	0.92 ± 0.06	0.96 ± 0.10	0.96	0.648	0.689
	**PC-O 38:5**	D	0.51 ± 0.06	0.69 ± 0.05	1.37	0.045	0.63 ± 0.05	0.57 ± 0.07	1.10	0.287	0.874
	**PC-O 42:6**	S	0.15 ± 0.01	0.16 ± 0.01	1.07	0.280	0.18 ± 0.01	0.13 ± 0.01	1.39	0.004	0.522
	**PC-O 44:6**	D	0.14 ± 0.01	0.10 ± 0.01	0.69	0.004	0.12 ± 0.01	0.12 ± 0.01	1.01	0.550	0.954
	**PE 36:2**	D	1.75 ± 0.13	2.89 ± 0.22	1.66	0.000	2.40 ± 0.28	2.24 ± 0.20	1.07	0.336	0.874
	**PE 36:4**	S	1.28 ± 0.10	1.05 ± 0.07	0.82	0.116	0.95 ± 0.07	1.38 ± 0.07	0.69	0.004	0.993
	**PE 38:4**	D	3.02 ± 0.12	4.11 ± 0.24	1.36	0.004	3.39 ± 0.24	3.73 ± 0.23	0.91	0.689	0.973
	**SM 18:1_2/14:0**	S	0.86 ± 0.09	0.75 ± 0.06	0.87	0.298	0.61 ± 0.03	1.00 ± 0.07	0.61	0.000	0.973
	**SM 18:1_2/15:0**	S	0.74 ± 0.05	0.80 ± 0.06	1.08	0.299	0.62 ± 0.03	0.92 ± 0.04	0.67	0.002	0.847
	**SM 18:1_2/16:0**	S	33.31 ± 4.22	31.45 ± 3.87	0.94	0.813	20.78 ± 1.37	43.98 ± 3.21	0.47	0.000	0.858
	**SM 18:1_2/17:0**	D	0.34 ± 0.02	0.50 ± 0.03	1.46	0.002	0.41 ± 0.03	0.43 ± 0.03	0.95	0.974	0.973
	**SM 18:1_2/18:0**	D	4.73 ± 0.37	8.04 ± 0.69	1.70	0.001	7.07 ± 0.80	5.70 ± 0.56	1.24	0.126	0.973
	**SM 18:1_2/18:1**	D	1.15 ± 0.06	1.73 ± 0.12	1.51	0.001	1.53 ± 0.14	1.35 ± 0.10	1.13	0.170	0.973
	**SM 18:1_2/20:0**	D	1.73 ± 0.12	2.32 ± 0.14	1.34	0.034	2.09 ± 0.13	1.96 ± 0.16	1.07	0.371	0.968
	**SM 18:1_2/20:3**	D	0.50 ± 0.06	0.99 ± 0.11	2.00	0.001	0.82 ± 0.14	0.67 ± 0.08	1.24	0.321	0.954
	**SM 18:1_2/20:4**	D	0.23 ± 0.01	0.32 ± 0.02	1.37	0.008	0.29 ± 0.02	0.26 ± 0.02	1.13	0.174	0.973
	**SM 18:1_2/22:1**	S	2.00 ± 0.12	1.83 ± 0.12	0.91	0.451	2.25 ± 0.08	1.57 ± 0.07	1.43	0.001	0.954
	**SM 18:1_2/23:1**	S	1.32 ± 0.08	1.20 ± 0.06	0.91	0.480	1.43 ± 0.06	1.09 ± 0.04	1.31	0.008	0.636
	**SM 18:1_2/24:0**	D	27.27 ± 1.31	20.76 ± 0.84	0.76	0.008	23.44 ± 1.65	24.58 ± 1.13	0.95	0.942	0.636
	**SM 18:1_2/25:0**	S	2.26 ± 0.13	1.95 ± 0.14	0.86	0.152	1.74 ± 0.10	2.47 ± 0.10	0.70	0.004	0.847
	**SM 36:0**	D	0.27 ± 0.03	0.45 ± 0.05	1.68	0.033	0.34 ± 0.04	0.37 ± 0.06	0.91	0.911	0.968

	**TG 48:0**	D	4.24 ± 0.82	2.19 ± 0.25	0.52	0.001	2.68 ± 0.28	3.74 ± 0.88	0.72	0.562	0.644
	**TG 48:1**	D	13.61 ± 3.65	4.53 ± 0.78	0.33	0.000	6.23 ± 0.82	11.91 ± 3.90	0.52	0.373	0.636
	**TG 48:2**	D	9.64 ± 3.11	1.99 ± 0.31	0.21	0.000	3.30 ± 0.60	8.34 ± 3.27	0.40	0.187	0.479
	**TG 48:3**	D	2.08 ± 0.57	0.62 ± 0.06	0.30	0.000	0.86 ± 0.11	1.84 ± 0.60	0.47	0.179	0.272
	**TG 50:0**	D	1.18 ± 0.18	2.59 ± 0.30	2.19	0.000	2.01 ± 0.37	1.76 ± 0.24	1.14	0.496	0.619
	**TG 50:2**	D	61.14 ± 13.63	24.06 ± 4.46	0.39	0.000	28.19 ± 3.81	57.01 ± 14.56	0.49	0.116	0.701
	**TG 50:3**	D	27.41 ± 7.28	6.47 ± 1.02	0.24	0.000	9.42 ± 1.49	24.46 ± 7.74	0.39	0.084	0.479
	**TG 50:4**	D	4.91 ± 1.27	1.20 ± 0.15	0.24	0.000	1.76 ± 0.25	4.35 ± 1.36	0.41	0.105	0.445
	**TG 51:1**	D	0.55 ± 0.07	1.02 ± 0.09	1.87	0.000	0.73 ± 0.12	0.84 ± 0.09	0.86	0.174	0.170
	**TG 51:4**	S	0.69 ± 0.13	0.34 ± 0.03	0.50	0.000	0.35 ± 0.02	0.68 ± 0.14	0.51	0.004	0.208
	**TG 52:0**	D	0.27 ± 0.03	2.08 ± 0.30	7.62	0.000	1.38 ± 0.40	0.97 ± 0.23	1.43	0.359	0.619
	**TG 52:1**	D	4.19 ± 0.70	14.18 ± 1.87	3.39	0.000	10.61 ± 2.34	7.76 ± 1.43	1.37	0.170	0.636
	**TG 52:2**	D	70.26 ± 14.04	94.55 ± 11.16	1.35	0.034	69.05 ± 9.26	95.76 ± 15.21	0.72	0.361	0.689
	**TG 52:4**	D	54.92 ± 11.61	25.23 ± 3.06	0.46	0.004	26.38 ± 2.88	53.77 ± 11.87	0.49	0.058	0.581
	**TG 52:5**	D	12.90 ± 2.83	3.85 ± 0.51	0.30	0.000	5.19 ± 0.66	11.55 ± 3.07	0.45	0.053	0.533
	**TG 53:3**	D	1.05 ± 0.18	1.56 ± 0.16	1.49	0.001	1.04 ± 0.13	1.56 ± 0.20	0.67	0.050	0.479
	**TG 54:1**	D	0.92 ± 0.10	3.94 ± 0.52	4.30	0.000	2.83 ± 0.68	2.02 ± 0.39	1.40	0.286	0.479
	**TG 54:2**	D	3.46 ± 0.47	11.78 ± 1.23	3.40	0.000	8.07 ± 1.67	7.18 ± 1.25	1.12	0.579	0.547
	**TG 54:3**	D	16.86 ± 3.29	31.66 ± 3.86	1.88	0.000	20.02 ± 3.20	28.50 ± 4.60	0.70	0.150	0.619
	**TG 54:6**	S	14.95 ± 2.68	11.05 ± 1.45	0.74	0.124	9.00 ± 0.69	17.01 ± 2.63	0.53	0.004	0.943
	**TG 54:7**	D; S	7.95 ± 1.61	2.09 ± 0.30	0.26	0.000	3.24 ± 0.55	6.79 ± 1.79	0.48	0.017	0.769
	**TG 56:3**	D	1.40 ± 0.20	2.28 ± 0.23	1.63	0.002	1.66 ± 0.22	2.02 ± 0.26	0.82	0.520	0.636
	**TG 56:4**	D	2.76 ± 0.35	4.01 ± 0.40	1.45	0.002	2.96 ± 0.32	3.81 ± 0.46	0.78	0.232	0.700
	**TG 56:5**	D; S	7.53 ± 1.16	12.20 ± 1.31	1.62	0.000	7.92 ± 0.90	11.81 ± 1.58	0.67	0.036	0.932
	**TG 56:6**	D; S	13.17 ± 2.44	18.63 ± 2.32	1.42	0.002	11.25 ± 1.11	20.55 ± 2.82	0.55	0.002	0.954
	**TG 56:7**	S	8.21 ± 1.57	8.55 ± 1.22	1.04	0.411	5.93 ± 0.47	10.83 ± 1.68	0.55	0.004	0.737
	**TG 56:8**	D; S	8.87 ± 1.77	5.58 ± 0.85	0.63	0.014	4.20 ± 0.36	10.26 ± 1.65	0.41	0.000	0.701
	**TG 58:8**	D; S	4.01 ± 0.75	5.58 ± 0.72	1.39	0.001	3.21 ± 0.31	6.39 ± 0.83	0.50	0.000	0.701
	**TG 58:9**	D; S	3.41 ± 0.53	4.45 ± 0.45	1.30	0.008	2.87 ± 0.28	4.99 ± 0.52	0.57	0.001	0.809
**Feces**	**ACar 18:0**	D	1.50 ± 0.22	6.26 ± 0.80	4.17	0.000	2.86 ± 0.60	4.90 ± 1.02	0.58	0.534	0.971
	**ACar 20:0**	D	0.47 ± 0.07	1.15 ± 0.14	2.43	0.025	0.71 ± 0.15	0.90 ± 0.13	0.15	0.927	0.980
	**TG 50:3**	D	18.95 ± 3.43	11.22 ± 2.43	0.59	0.042	13.96 ± 2.53	16.21 ± 3.65	0.86	0.752	0.876

	**TG 52:1**	D	0.63 ± 0.24	16.85 ± 5.99	26.55	0.001	14.62 ± 6.29	2.86 ± 1.10	5.11	0.814	0.590
	**TG 53:3**	D	5.46 ± 0.80	2.22 ± 0.34	0.41	0.002	4.11 ± 0.56	3.57 ± 0.92	1.15	0.410	0.959
	**TG 54:7**	D	6.19 ± 1.23	2.01 ± 0.39	0.32	0.000	3.58 ± 0.57	4.63 ± 1.40	0.77	0.612	0.590

Data are expressed by mean ± SEM (n=14 per group and sex). FC: fold change; D: diet effect; S: sex effect.

Table S7. Differential plasma lipid species across comparisons.

					Females *vs* Males					HFD *vs* REF				
	REF		HFD		REF		HFD			Females		Males		
**Lipid species**	**Females**	**Males**	**Females**	**Males**	**FC**	***p*-adj**	**FC**	***p*-adj**	**Direction consistency**	**FC**	***p*-adj**	**FC**	***p*-adj**	**Direction consistency**
**ChoE 14:0**	1.13 ± 0.12	1.93 ± 0.33	0.44 ± 0.04	0.98 ± 0.22	0.659	0.028	0.491	0.009	Same	0.394	0.000	-	-	-
**ChoE 16:1**	38.12 ± 3.78	84.25 ± 10.06	13.30 ± 0.79	26.46 ± 4.31	0.509	0.012	0.529	0.007	Same	0.341	0.000	0.327	0.000	Same
**ChoE 18:0**	2.95 ± 0.20	3.12 ± 0.47	6.37 ± 0.47	6.62 ± 0.63	-	-	-	-	-	2.134	0.000	2.295	0.001	Same
**ChoE 18:1**	104.12 ± 4.21	161.03 ± 11.81	140.31 ± 8.23	325.81 ± 29.66	-	-	0.442	0.000	-	-	-	2.137	0.000	-
**ChoE 18:3**	151.76 ± 13.28	145.63 ± 19.34	72.96 ± 5.68	84.22 ± 10.01	-	-	-	-	-	0.472	0.000	0.591	0.017	Same
**ChoE 20:5**	464.42 ± 39.26	335.70 ± 41.16	133.69 ± 13.82	90.42 ± 7.54	-	-	1.506	0.048	-	0.283	0.000	0.277	0.000	Same
**DG 34:1**	0.90 ± 0.07	1.29 ± 0.16	1.51 ± 0.17	1.52 ± 0.23	-	-	-	-	-	1.606	0.002	-	-	-
**DG 36:2**	1.19 ± 0.16	1.79 ± 0.26	1.87 ± 0.18	2.52 ± 0.47	-	-	-	-	-	1.534	0.006	-	-	-
**LPC 14:0**	1.13 ± 0.06	1.48 ± 0.16	0.60 ± 0.05	0.69 ± 0.08	-	-	-	-	-	0.517	0.000	0.489	0.000	Same
**LPC 15:0**	0.67 ± 0.02	0.80 ± 0.06	0.43 ± 0.02	0.48 ± 0.03	-	-	-	-	-	0.636	0.001	0.63	0.003	Same
**LPC 16:0**	94.67 ± 5.35	106.10 ± 8.78	61.21 ± 5.23	74.43 ± 4.98	-	-	-	-	-	0.643	0.001	0.606	0.001	Same
**LPC 16:1**	4.55 ± 0.48	7.46 ± 1.29	1.52 ± 0.09	1.81 ± 0.32	-	-	-	-	-	0.328	0.000	0.258	0.000	Same
**LPC 18:0**	114.62 ± 7.56	71.19 ± 6.63	118.03 ± 6.52	93.45 ± 6.62	1.742	0.017	-	-	-	-	-	-	-	-
**LPC 18:2**	49.57 ± 4.92	53.60 ± 7.91	39.12 ± 4.27	30.92 ± 4.92	-	-	-	-	-	-	-	0.608	0.023	-
**LPC 18:3**	10.66 ± 0.98	13.43 ± 1.68	5.59 ± 0.56	7.46 ± 0.69	-	-	-	-	-	0.53	0.000	0.596	0.007	Same
**LPC 19:0**	0.61 ± 0.04	0.60 ± 0.03	0.40 ± 0.01	0.44 ± 0.04	-	-	-	-	-	0.655	0.000	-	-	-
**LPC 20:0**	0.45 ± 0.02	0.35 ± 0.02	0.27 ± 0.01	0.28 ± 0.02	-	-	-	-	-	0.603	0.001	-	-	-
**LPC 20:3**	1.99 ± 0.27	2.68 ± 0.52	1.26 ± 0.12	1.37 ± 0.19	-	-	-	-	-	0.628	0.040	0.552	0.014	Same
**LPC 20:5**	4.67 ± 0.53	5.59 ± 1.07	3.40 ± 0.39	2.97 ± 0.49	-	-	-	-	-	-	-	0.569	0.037	-
**LPC 22:5**	0.51 ± 0.06	0.57 ± 0.09	0.31 ± 0.03	0.33 ± 0.04	-	-	-	-	-	0.605	0.014	0.62	0.034	Same
**LPC 24:0**	0.31 ± 0.01	0.28 ± 0.01	0.19 ± 0.01	0.20 ± 0.01	-	-	-	-	-	0.609	0.000	-	-	-
**LPE 16:0**	1.87 ± 0.13	1.58 ± 0.10	1.34 ± 0.12	0.91 ± 0.05	-	-	1.504	0.012	-	-	-	-	-	-
**LPE 18:2**	1.26 ± 0.09	0.98 ± 0.18	1.25 ± 0.17	0.73 ± 0.09	-	-	1.745	0.020	-	-	-	-	-	-
**PC 30:0**	0.88 ± 0.05	1.07 ± 0.12	0.52 ± 0.05	0.52 ± 0.02	-	-	-	-	-	0.583	0.000	0.517	0.003	Same
**PC 30:1**	0.28 ± 0.03	0.42 ± 0.08	0.11 ± 0.01	0.15 ± 0.01	-	-	-	-	-	0.385	0.000	0.4	0.000	Same

**PC 31:0**	0.46 ± 0.02	0.50 ± 0.05	0.26 ± 0.01	0.26 ± 0.01	-	-	-	-	-	0.55	0.000	0.55	0.006	Same
**PC 32:1**	6.46 ± 0.65	12.35 ± 2.46	2.18 ± 0.17	3.08 ± 0.42	-	-	-	-	-	0.331	0.000	0.273	0.000	Same
**PC 32:2**	1.30 ± 0.14	2.42 ± 0.41	0.58 ± 0.06	0.83 ± 0.09	0.61	0.028	-	-	-	0.441	0.000	0.374	0.000	Same
**PC 33:2**	0.88 ± 0.05	1.19 ± 0.18	0.55 ± 0.07	0.67 ± 0.05	-	-	-	-	-	0.609	0.000	0.6	0.013	Same
**PC 34:1**	49.34 ± 3.23	64.83 ± 8.70	40.61 ± 3.20	63.06 ± 4.18	-	-	0.658	0.003	-	-	-	-	-	-
**PC 34:3**	4.41 ± 0.44	6.84 ± 1.19	2.24 ± 0.24	2.75 ± 0.21	-	-	-	-	-	0.498	0.000	0.441	0.001	Same
**PC 34:4**	0.85 ± 0.10	1.99 ± 0.35	0.42 ± 0.03	1.01 ± 0.19	0.492	0.012	0.443	0.001	Same	0.484	0.000	0.537	0.006	Same
**PC 34:5**	0.14 ± 0.01	0.27 ± 0.04	0.06 ± 0.01	0.10 ± 0.01	0.589	0.019	0.61	0.006	Same	0.435	0.000	0.421	0.000	Same
**PC 35:4**	0.21 ± 0.02	0.33 ± 0.05	0.17 ± 0.01	0.26 ± 0.02	-	-	0.651	0.008	-	-	-	-	-	-
**PC 36:1**	22.68 ± 1.52	15.52 ± 2.16	23.25 ± 1.52	23.45 ± 1.88	1.608	0.028	-	-	-	-	-	1.576	0.017	-
**PC 36:3**	38.69 ± 3.88	60.64 ± 7.34	28.87 ± 2.44	44.53 ± 3.33	-	-	0.665	0.001	-	-	-	-	-	-
**PC 36:4**	138.46 ± 13.97	197.15 ± 17.35	92.40 ± 7.90	189.47 ± 11.94	-	-	0.496	0.000	-	0.665	0.001	-	-	-
**PC 36:5**	17.47 ± 2.02	21.80 ± 2.87	10.66 ± 1.60	14.73 ± 0.80	-	-	-	-	-	0.6	0.003	-	-	-
**PC 37:2**	1.22 ± 0.10	1.25 ± 0.13	0.72 ± 0.04	0.88 ± 0.07	-	-	-	-	-	0.591	0.000	-	-	-
**PC 37:4**	2.92 ± 0.32	3.34 ± 0.22	2.41 ± 0.16	4.35 ± 0.36	-	-	0.577	0.004	-	-	-	-	-	-
**PC 38:5**	12.01 ± 1.61	30.20 ± 5.01	8.45 ± 0.61	19.45 ± 2.42	0.445	0.012	0.456	0.000	Same	-	-	-	-	-
**PC 38:6**	46.34 ± 5.88	61.39 ± 7.91	32.81 ± 5.05	58.93 ± 4.22	-	-	0.565	0.008	-	-	-	-	-	-
**PC 38:7**	14.38 ± 1.54	21.85 ± 1.94	9.00 ± 0.83	20.50 ± 1.43	-	-	0.446	0.000	-	0.624	0.001	-	-	-
**PC 40:4**	2.10 ± 0.12	1.25 ± 0.16	2.05 ± 0.05	1.89 ± 0.15	1.832	0.012	-	-	-	-	-	1.583	0.021	-
**PC 40:6**	88.93 ± 8.78	40.77 ± 6.39	82.24 ± 6.75	51.85 ± 4.57	2.308	0.017	1.64	0.006	Same	-	-	-	-	-
**PC 40:9**	5.01 ± 0.59	6.15 ± 0.62	3.72 ± 0.54	6.08 ± 0.40	-	-	0.617	0.020	-	-	-	-	-	-
**PC-O 38:4**	0.76 ± 0.08	0.71 ± 0.11	1.08 ± 0.05	1.20 ± 0.12	-	-	-	-	-	-	-	1.763	0.036	-
**PC-O 42:6**	0.19 ± 0.01	0.12 ± 0.02	0.18 ± 0.01	0.15 ± 0.01	1.718	0.028	-	-	-	-	-	-	-	-
**PE 36:2**	1.70 ± 0.17	1.79 ± 0.22	3.10 ± 0.39	2.68 ± 0.23	-	-	-	-	-	1.779	0.001	1.58	0.033	Same
**SM 18:1;2/14:0**	0.64 ± 0.05	1.09 ± 0.11	0.58 ± 0.04	0.91 ± 0.07	-	-	0.651	0.011	-	-	-	-	-	-
**SM 18:1;2/15:0**	0.60 ± 0.04	0.88 ± 0.06	0.63 ± 0.03	0.97 ± 0.05	-	-	0.664	0.020	-	-	-	-	-	-
**SM 18:1;2/16:0**	22.17 ± 2.59	44.45 ± 5.39	19.39 ± 0.85	43.50 ± 3.95	-	-	0.448	0.006	-	-	-	-	-	-
**SM 18:1;2/18:0**	5.11 ± 0.57	4.34 ± 0.45	9.02 ± 1.08	7.06 ± 0.74	-	-	-	-	-	1.72	0.003	-	-	-

**SM 18:1;2/18:1**	1.18 ± 0.09	1.11 ± 0.09	1.87 ± 0.19	1.59 ± 0.14	-	-	-	-	-	1.545	0.000	-	-	-
**SM 18:1;2/20:3**	0.55 ± 0.10	0.45 ± 0.07	1.10 ± 0.21	0.89 ± 0.08	-	-	-	-	-	1.973	0.025	2.149	0.024	Same
**SM 18:1;2/22:1**	2.36 ± 0.13	1.63 ± 0.07	2.14 ± 0.10	1.51 ± 0.13	1.555	0.017	-	-	-	-	-	-	-	-
**SM 18:1;2/23:1**	1.55 ± 0.07	1.09 ± 0.05	1.31 ± 0.08	1.09 ± 0.06	1.521	0.026	-	-	-	-	-	-	-	-
**TG 48:0**	3.20 ± 0.39	5.27 ± 1.55	2.17 ± 0.31	2.21 ± 0.43	-	-	-	-	-	-	-	0.463	0.018	-
**TG 48:1**	8.27 ± 1.12	18.95 ± 6.86	4.20 ± 0.53	4.86 ± 1.52	-	-	-	-	-	0.483	0.007	0.281	0.014	Same
**TG 48:2**	4.72 ± 0.92	14.57 ± 5.75	1.87 ± 0.22	2.11 ± 0.59	-	-	-	-	-	0.383	0.003	0.164	0.004	Same
**TG 48:3**	1.08 ± 0.18	3.08 ± 1.02	0.64 ± 0.07	0.60 ± 0.11	-	-	-	-	-	0.575	0.023	0.221	0.004	Same
**TG 50:0**	1.05 ± 0.17	1.32 ± 0.33	2.98 ± 0.50	2.20 ± 0.29	-	-	-	-	-	2.607	0.002	1.85	0.019	Same
**TG 50:2**	36.96 ± 5.64	85.31 ± 24.05	19.42 ± 2.34	28.70 ± 8.58	-	-	-	-	-	0.513	0.005	0.359	0.037	Same
**TG 50:3**	13.14 ± 2.13	41.67 ± 12.54	5.70 ± 0.66	7.24 ± 1.97	-	-	-	-	-	0.427	0.001	0.188	0.006	Same
**TG 50:4**	2.38 ± 0.36	7.45 ± 2.18	1.15 ± 0.12	1.25 ± 0.28	-	-	-	-	-	0.477	0.002	0.185	0.004	Same
**TG 51:1**	0.39 ± 0.05	0.71 ± 0.11	1.06 ± 0.13	0.98 ± 0.13	-	-	-	-	-	2.584	0.000	-	-	-
**TG 51:4**	0.38 ± 0.03	1.00 ± 0.21	0.32 ± 0.03	0.37 ± 0.06	0.437	0.028	-	-	-	-	-	-	-	-
**TG 52:0**	0.25 ± 0.03	0.29 ± 0.05	2.51 ± 0.51	1.64 ± 0.28	-	-	-	-	-	9.164	0.000	5.891	0.000	Same
**TG 52:1**	4.00 ± 0.77	4.37 ± 1.23	17.21 ± 2.92	11.14 ± 1.89	-	-	-	-	-	3.95	0.000	2.838	0.004	Same
**TG 52:2**	49.28 ± 10.07	91.24 ± 24.62	88.83 ± 11.81	100.28 ± 19.73	-	-	-	-	-	1.726	0.026	-	-	-
**TG 52:4**	30.45 ± 4.53	79.38 ± 19.08	22.31 ± 3.15	28.16 ± 5.27	-	-	-	-	-	-	-	0.378	0.045	-
**TG 52:5**	6.98 ± 0.80	18.81 ± 4.74	3.40 ± 0.43	4.29 ± 0.93	-	-	-	-	-	0.48	0.000	0.244	0.006	Same
**TG 53:3**	0.66 ± 0.07	1.43 ± 0.29	1.43 ± 0.16	1.69 ± 0.28	-	-	-	-	-	2.094	0.000	-	-	-
**TG 54:1**	0.83 ± 0.13	1.01 ± 0.16	4.83 ± 0.80	3.04 ± 0.54	-	-	-	-	-	5.436	0.000	3.231	0.006	Same
**TG 54:2**	2.92 ± 0.34	4.01 ± 0.87	13.22 ± 1.78	10.35 ± 1.65	-	-	-	-	-	4.316	0.000	2.807	0.004	Same
**TG 54:3**	10.80 ± 1.12	22.92 ± 5.79	29.24 ± 3.86	34.07 ± 6.90	-	-	-	-	-	2.614	0.000	-	-	-
**TG 54:4**	19.03 ± 2.10	43.22 ± 10.44	31.30 ± 3.62	40.53 ± 7.39	-	-	-	-	-	1.617	0.004	-	-	-
**TG 54:7**	4.85 ± 0.66	11.04 ± 2.76	1.63 ± 0.16	2.54 ± 0.55	-	-	-	-	-	0.334	0.000	0.247	0.003	Same
**TG 56:3**	1.08 ± 0.15	1.71 ± 0.33	2.23 ± 0.27	2.34 ± 0.39	-	-	-	-	-	1.985	0.003	-	-	-
**TG 56:4**	2.17 ± 0.25	3.34 ± 0.59	3.75 ± 0.44	4.28 ± 0.70	-	-	-	-	-	1.673	0.009	-	-	-
**TG 56:5**	5.53 ± 0.42	9.53 ± 2.09	10.30 ± 1.21	14.10 ± 2.18	-	-	-	-	-	1.782	0.004	1.599	0.042	Same

**TG 56:6**	8.46 ± 0.56	17.87 ± 4.26	14.03 ± 1.56	23.23 ± 3.73	-	-	0.646	0.042	-	1.603	0.007	-	-	-
**TG 56:8**	4.67 ± 0.56	13.08 ± 2.71	3.73 ± 0.41	7.44 ± 1.36	0.412	0.019	0.533	0.006	Same	-	-	-	-	-
**TG 58:2**	0.53 ± 0.07	0.67 ± 0.10	0.82 ± 0.10	0.61 ± 0.10	-	-	-	-	-	1.501	0.042	-	-	-
**TG 58:8**	2.35 ± 0.23	5.68 ± 1.20	4.06 ± 0.35	7.10 ± 1.16	0.483	0.027	0.612	0.008	Same	1.68	0.003	-	-	-
**TG 58:9**	2.27 ± 0.30	4.56 ± 0.82	3.47 ± 0.37	5.43 ± 0.66	-	-	0.666	0.023	-	1.502	0.030	-	-	-

Data are expressed by mean ± SEM (n=7 per group and sex). FC: fold change.

Table S8. Significant FDR-adjusted Spearman correlations between lipid species and physiological variables within dietary groups.

	Group	Lipid class	Lipid species	Variable	ϱ	*p*-adj
Plasma	REF	SM	SM 18:1;2/23:1	BMI	-0.793	0.045
			SM 18:1;2/14:0	Leptin	0.837	0.030
			SM 18:1;2/14:0	Leptin/adiponectin ratio	0.864	0.022
			SM 18:1;2/15:0	Leptin/adiponectin ratio	0.802	0.044
			SM 18:1;2/16:0	Leptin/adiponectin ratio	0.820	0.035
	HFD	ChoE	ChoE 16:1	BMI	0.754	0.038
			ChoE 14:0	BW gain	0.763	0.038
			ChoE 16:1	BW gain	0.776	0.038
			ChoE 18:1	BW gain	0.736	0.044
			ChoE 14:0	Leptin/adiponectin ratio	0.749	0.038
			ChoE 14:0	WC	0.756	0.038
			ChoE 16:1	WC	0.815	0.038
			ChoE 18:1	WC	0.756	0.038
	REF	ChoE	ChoE 16:1	BMI	0.754	0.024
			ChoE 18:1	BMI	0.776	0.021
			ChoE 14:0	BW gain	0.763	0.022
			ChoE 18:1	BW gain	0.807	0.016
			ChoE 16:0	Leptin	0.842	0.010
			ChoE 16:1	Leptin	0.727	0.038
			ChoE 16:0	Leptin/adiponectin ratio	0.837	0.010
			ChoE 16:1	Leptin/adiponectin ratio	0.780	0.021
			ChoE 18:1	Leptin/adiponectin ratio	0.767	0.022
			ChoE 16:1	WC	0.775	0.021
			ChoE 18:1	WC	0.830	0.010
	REF	DG	DG 34:1	BW gain	0.802	0.037
	HFD	LPE	LPE 18:2	Adiponectin	0.824	0.041
	REF	PE	PE 36:4	Leptin/adiponectin ratio	0.877	0.005
	HFD	PC	PC 34:4	BMI	0.776	0.049
			PC 34:5	BMI	0.780	0.049
			PC 36:4	BMI	0.763	0.049
			PC 30:1	BW gain	0.763	0.049
			PC 34:4	BW gain	0.811	0.040
			PC 36:3	BW gain	0.767	0.049
			PC 36:4	BW gain	0.815	0.040
			PC 38:5	BW gain	0.785	0.049
			PC 38:7	BW gain	0.789	0.049
			PC 34:4	Leptin/adiponectin ratio	0.767	0.049
			PC 36:4	Leptin/adiponectin ratio	0.771	0.049
			PC 32:1	WC	0.765	0.049
			PC 34:4	WC	0.762	0.049
			PC 34:5	WC	0.782	0.049

			PC 35:4	WC	0.866	0.038
			PC 36:3	WC	0.815	0.040
			PC 36:4	WC	0.831	0.040
			PC 38:5	WC	0.809	0.040
			PC 38:7	WC	0.809	0.040
Feces	HFD	ACar	ACar 16:0	BW gain	0.859	0.009
			ACar 18:1	BW gain	0.798	0.035

Values are Spearman correlation coefficients (ϱ) and Benjamini–Hochberg FDR-adjusted *p*-values (*p*-adj). Only associations with *p*-adj < 0.05 are shown. HFD, high-fat diet; REF, reference diet; SM, sphingomyelin; ChoE, cholesteryl ester; DG, diglyceride; LPE, lysophosphatidylethanolamine; PE, phosphatidylethanolamine; PC, phosphatidylcholine; ACar, acylcarnitine; BMI, body mass index; BW, body weight; WC, waist circumference.

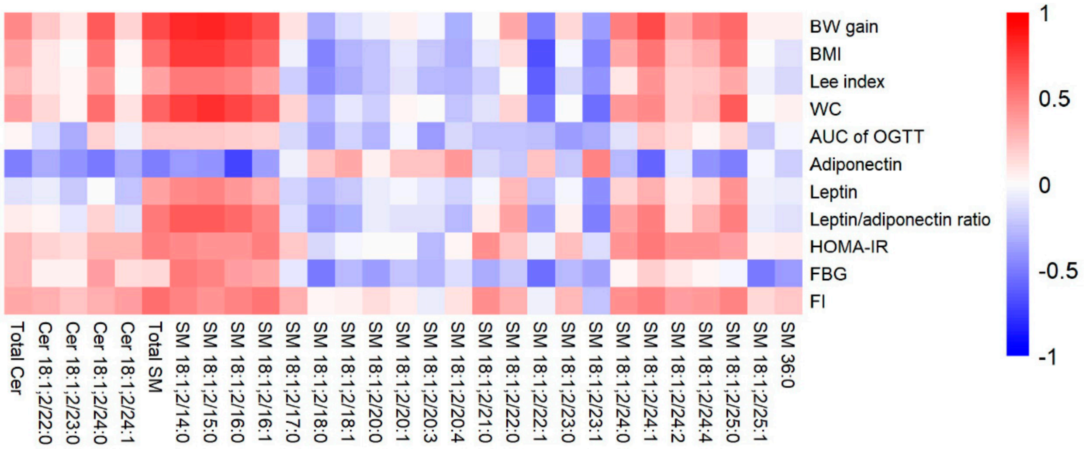

Figure S1. Plasma sphingolipid correlation study in the HFD group. Spearman correlations between ceramide (Cer) and sphingomyelin (SM) species and physiological variables in the HFD group (n = 14). Spearman correlation coefficients are represented by color, with blue indicating negative correlations and red indicating positive correlations. BW gain, body weight gain; BMI, body mass index; WC, waist circumference; AUC OGTT, area under the curve of the oral glucose tolerance test; HOMA-IR, homeostatic model assessment of insulin resistance; FBG, fasting blood glucose; FI, fasting insulin.

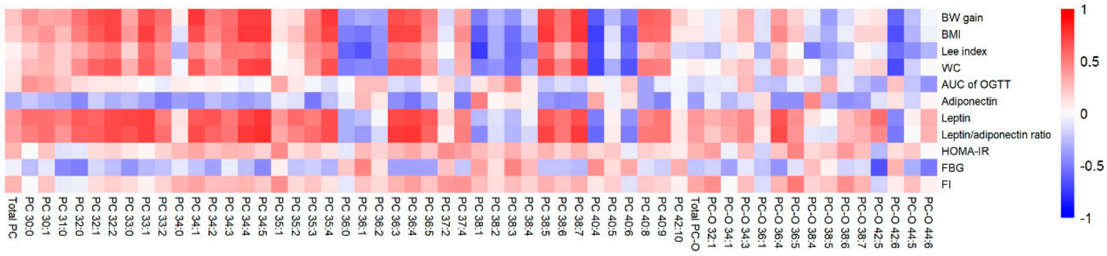

Figure S2. Plasma phosphatidylcholine (PC) correlation study in the HFD group. Spearman correlations between PC species and physiological variables in the REF group (n = 14). Spearman correlation coefficients are represented by color, with blue indicating negative correlations and red indicating positive correlations. BW gain, body weight gain; BMI, body mass index; WC, waist circumference; AUC OGTT, area under the curve of the oral glucose tolerance test; HOMA-IR, homeostatic model assessment of insulin resistance; FBG, fasting blood glucose; FI, fasting insulin.

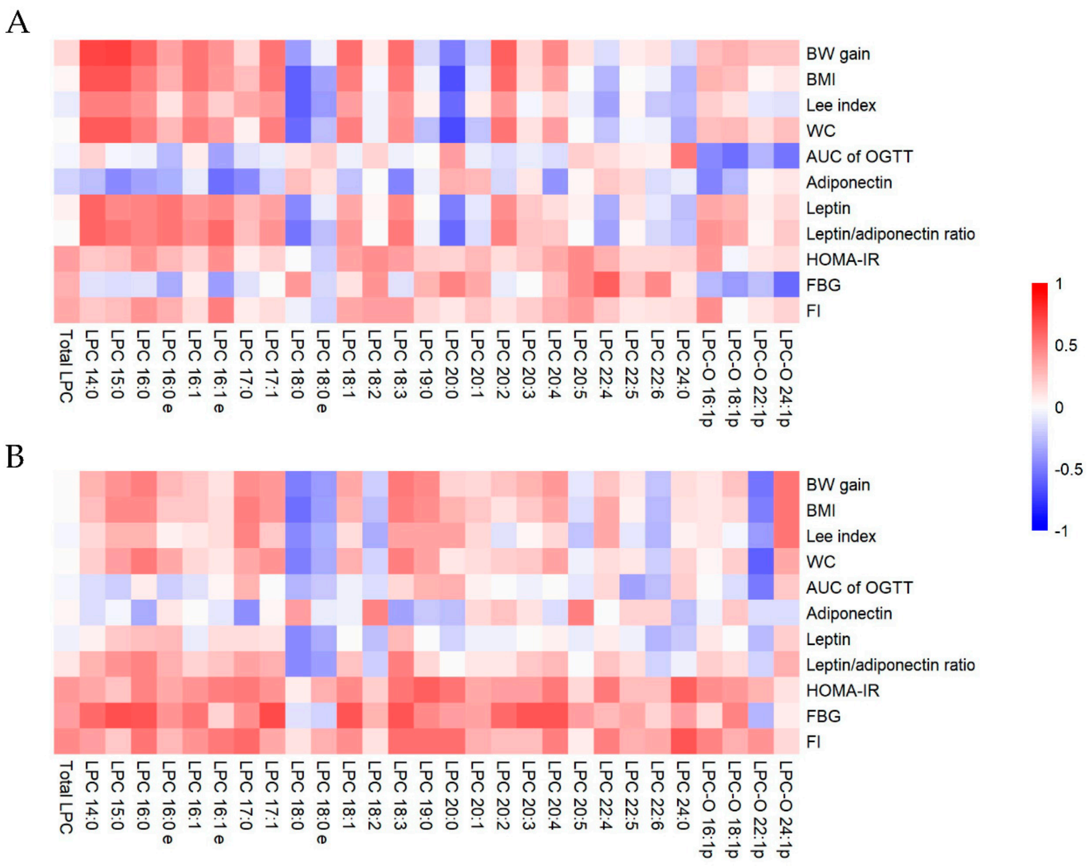

Figure S3. Plasma lysophosphatidylcholine (LPC) correlation study within dietary groups. Spearman correlations between LPC species and physiological variables in the (A) REF group (n = 14) and (B) HFD hroup (n = 14). Spearman correlation coefficients are represented by color, with blue indicating negative correlations and red indicating positive correlations. BW gain, body weight gain; BMI, body mass index; WC, waist circumference; AUC OGTT, area under the curve of the oral glucose tolerance test; HOMA-IR, homeostatic model assessment of insulin resistance; FBG, fasting blood glucose; FI, fasting insulin.

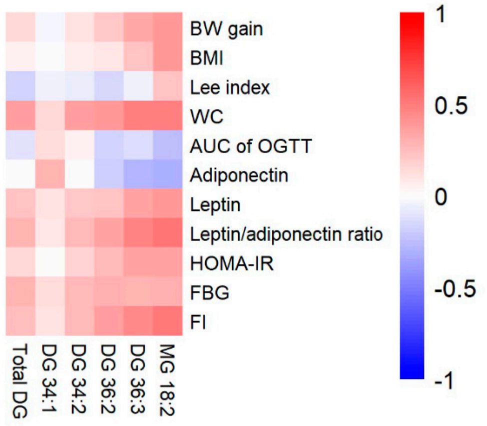

Figure S4. Plasma glycerolipid correlation study in the HFD group. Spearman correlations between monoacylglycerol (MG) and diacylglycerol (DG) species and physiological variables in the HFD group (n = 14). Spearman correlation coefficients are represented by color, with blue indicating negative correlations and red indicating positive correlations. BW gain, body weight gain; BMI, body mass index; WC, waist circumference; AUC OGTT, area under the curve of the oral glucose tolerance test; HOMA-IR, homeostatic model assessment of insulin resistance; FBG, fasting blood glucose; FI, fasting insulin.

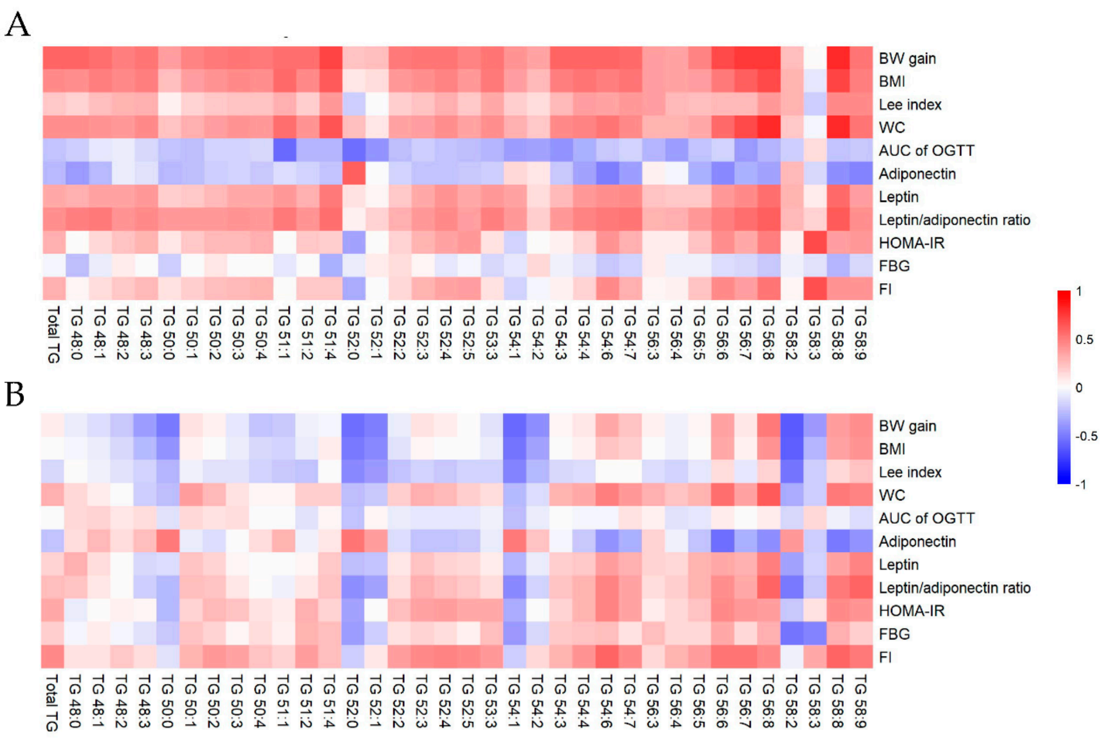

Figure S5. Plasma triacylglycerols (TG) correlation study within dietary groups. Spearman correlations between TG species and physiological variables in the (A) REF group (n = 14) and (B) HFD hroup (n = 14). Spearman correlation coefficients are represented by color, with blue indicating negative correlations and red indicating positive correlations. BW gain, body weight gain; BMI, body mass index; WC, waist circumference; AUC OGTT, area under the curve of the oral glucose tolerance test; HOMA-IR, homeostatic model assessment of insulin resistance; FBG, fasting blood glucose; FI, fasting insulin.

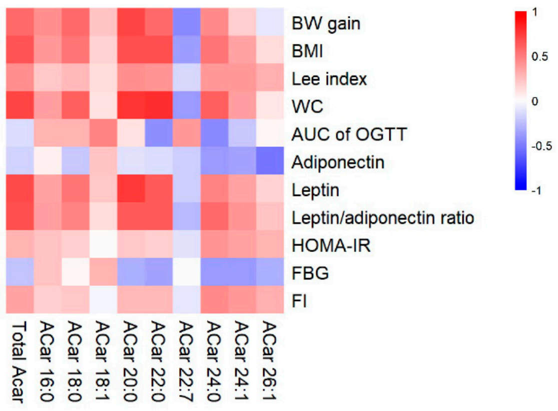

Figure S6. Fecal acylcarnitine (ACar) correlation study in the REF group. Spearman correlations between ACar species and physiological variables in the REF group (n = 14). Spearman correlation coefficients are represented by color, with blue indicating negative correlations and red indicating positive correlations. BW gain, body weight gain; BMI, body mass index; WC, waist circumference; AUC OGTT, area under the curve of the oral glucose tolerance test; HOMA-IR, homeostatic model assessment of insulin resistance; FBG, fasting blood glucose; FI, fasting insulin.

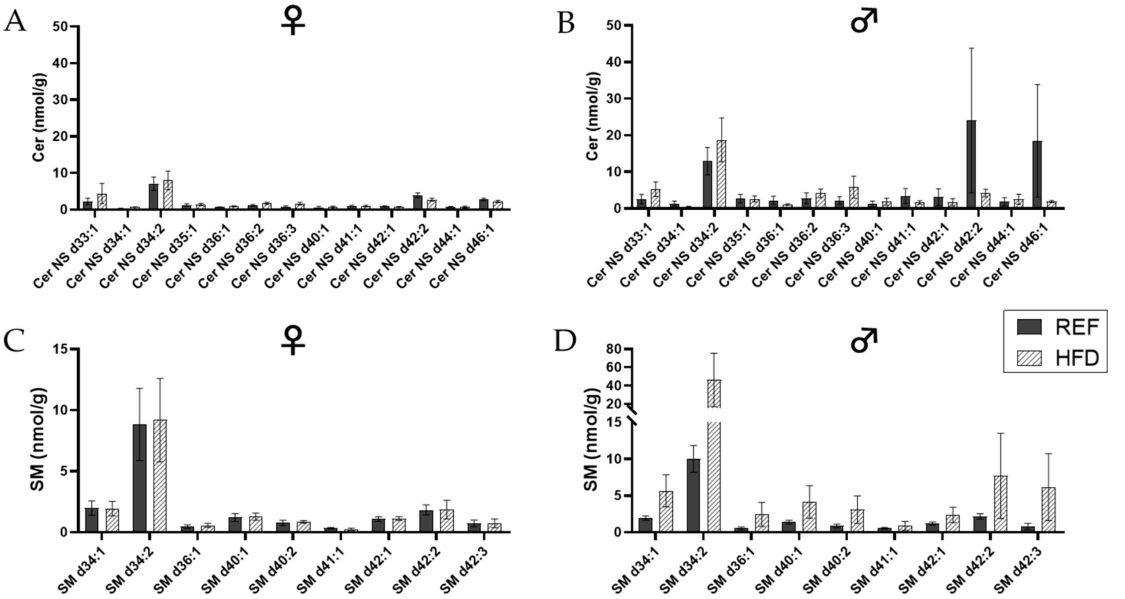

Figure S7. Fecal sphingolipids. Fecal concentration of detected ceramide (Cer) species in (A) female rats and (B) male rats; and detected sphingomyelin (SM) species in (C) female rats and (D) male rats. Data are expressed as mean ± SEM (n=7/group and sex). Fecal sphingolipids are reported according to lipid subclass and total carbon:number of double bonds reflecting the level of structural identification achieved.

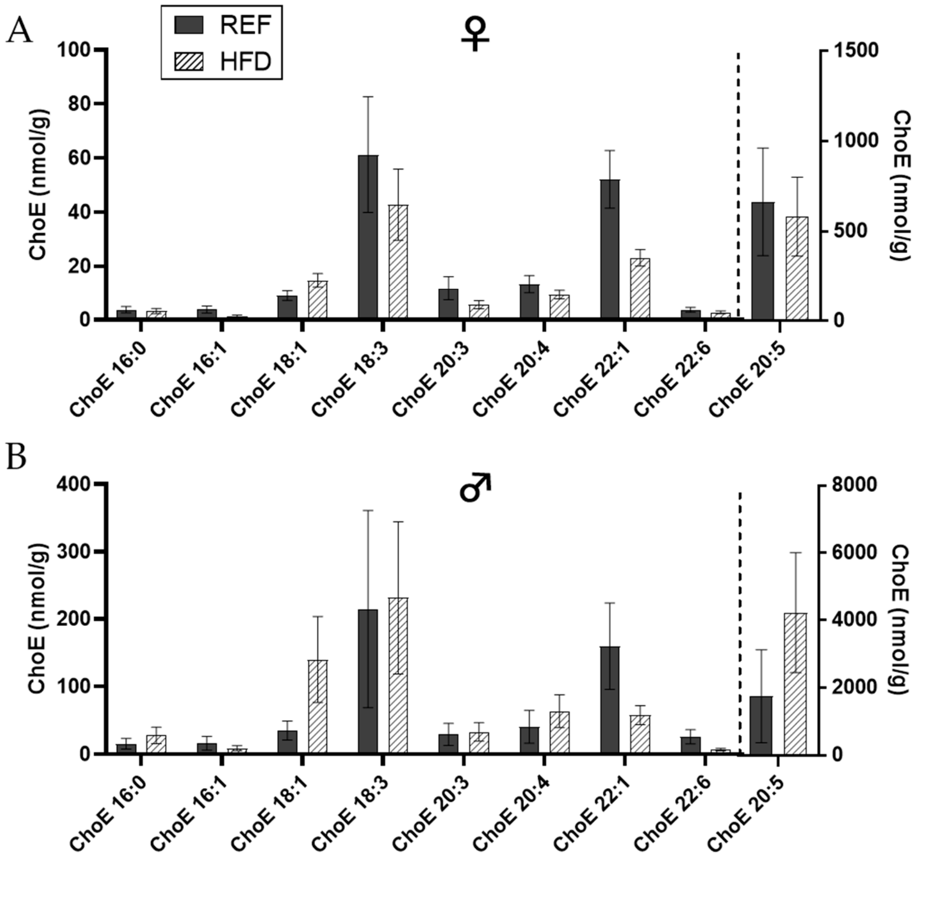

Figure S8. Fecal concentration of detected cholesteryl ester (ChoE) species in (A) female rats and (B) male rats. Data are expressed as mean ± SEM (n=7/group and sex).

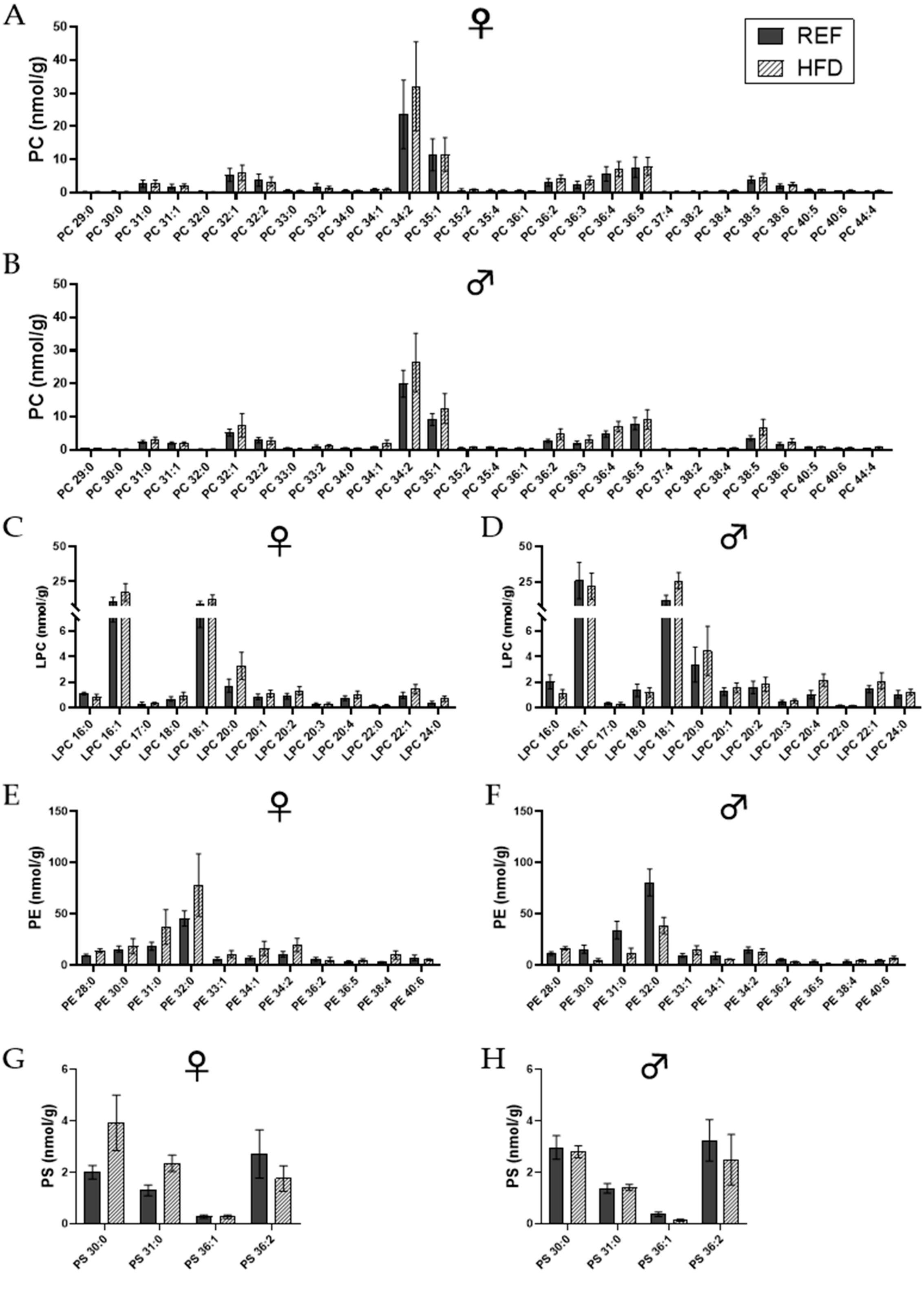

Figure S9. Fecal glycerophospholipids. Fecal concentration of detected phosphatidylcholines (PC) species in (A) female rats and (B) male rats; lysophosphatidylcholine (LPC) species in (C) female rats and (D) male rats; phosphatidylethanolamine (PE) species in (E) female rats and (F) male rats; phosphatidylserine (PS) species in (G) female rats and (H) male rats. Data are expressed as mean ± SEM (n=7/group and sex).

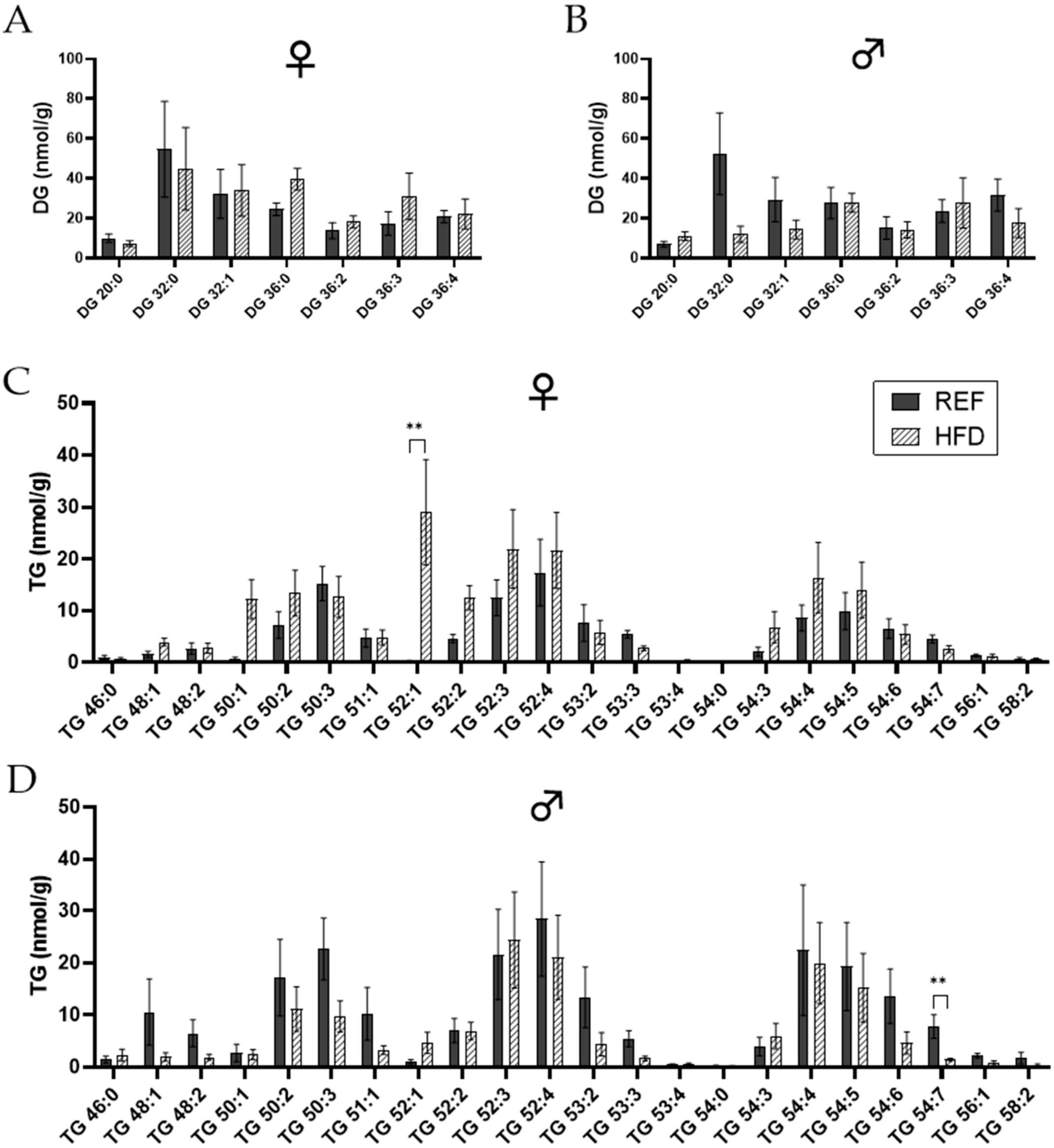

Figure S10. Fecal glycerolipids. Fecal concentration of detected diacylglycerol (DG) species in (A) female rats and (B) male rats; triacylglycerol (TG) species in (C) female rats and (D) male rats. Data are expressed as mean ± SEM. Statistical significance was assessed by t-test of transformed data with FDR correction (n=7/group and sex). Statistical difference: ** p-adj ≤ 0.01.
